# Gene Expression Profiling of Preovulatory Follicle in the Buffalo Cow: Effects of Increased IGF-I Concentration on Periovulatory Events

**DOI:** 10.1371/journal.pone.0020754

**Published:** 2011-06-20

**Authors:** Jyotsna U. Rao, Kunal B. Shah, Jayaram Puttaiah, Medhamurthy Rudraiah

**Affiliations:** Department of Molecular Reproduction, Development and Genetics, Indian Institute of Science, Bangalore, India; University of Muenster, Germany

## Abstract

The preovulatory follicle in response to gonadotropin surge undergoes dramatic biochemical, and morphological changes orchestrated by expression changes in hundreds of genes. Employing well characterized bovine preovulatory follicle model, granulosa cells (GCs) and follicle wall were collected from the preovulatory follicle before, 1, 10 and 22 h post peak LH surge. Microarray analysis performed on GCs revealed that 450 and 111 genes were differentially expressed at 1 and 22 h post peak LH surge, respectively. For validation, qPCR and immunocytochemistry analyses were carried out for some of the differentially expressed genes. Expression analysis of many of these genes showed distinct expression patterns in GCs and the follicle wall. To study molecular functions and genetic networks, microarray data was analyzed using Ingenuity Pathway Analysis which revealed majority of the differentially expressed genes to cluster within processes like steroidogenesis, cell survival and cell differentiation. In the ovarian follicle, IGF-I is established to be an important regulator of the above mentioned molecular functions. Thus, further experiments were conducted to verify the effects of increased intrafollicular IGF-I levels on the expression of genes associated with the above mentioned processes. For this purpose, buffalo cows were administered with exogenous bGH to transiently increase circulating and intrafollicular concentrations of IGF-I. The results indicated that increased intrafollicular concentrations of IGF-I caused changes in expression of genes associated with steroidogenesis (StAR, SRF) and apoptosis (BCL-2, FKHR, PAWR). These results taken together suggest that onset of gonadotropin surge triggers activation of various biological pathways and that the effects of growth factors and peptides on gonadotropin actions could be examined during preovulatory follicle development.

## Introduction

The buffalo cow is an economically important livestock species in many Asian, Mediterranean and Latin countries. Its genetic improvement, especially in reproductive performance ranks high among agricultural research needs of these countries. Several aspects of reproduction in the buffalo cows are reported to be similar to dairy cattle [1]. However, a better understanding of follicular growth and ovulation may be essential for enhancing fertilization rate and for minimizing early embryonic losses. The failures in conception often may arise due to defects in the process of transformation of follicular structure into luteal structure essential for maintenance of pregnancy in these animals. Several studies have reported identification of differentially expressed genes in the dominant follicle of cattle [2]–[4]. Around the time of deviation, the dominant follicle acquires increased LH receptor (LHR) expression and aromatase activity in granulosa cells (GCs) as well as augmentation in theca cell androgen production [5], [6]. Associated with the process of selection, a concordant increase in estradiol (E_2_) concentration occurs in the dominant follicle leading to gonadotropin surge necessary for ovulation [7]–[9]. The interval between the gonadotropin surge and ovulation is a time of dramatic biochemical and morphological changes leading to rupture and transformation of follicle into corpus luteum (CL). The complex series of events triggered by gonadotropin surge appear to be highly coordinated temporally. The gonadotropin surge-induced increase in the progesterone receptor (PR) expression has been suggested to play important roles by conferring resistance to Fas-mediated apoptosis and regulating expression of downstream genes like oxytocin and COX-2 [10]–[12]. In cattle, the gonadotropin surge-induced expression of COX-2 around the time of ovulation appears to be necessary for production of PGE_2_ and PGF_2α_, both of which are essential for cumulus expansion and follicular rupture [12], [13].

In order to examine various mechanisms and the signal transduction pathways through which gonadotropin surge mediate its actions, analysis of changes in the transcriptome of preovulatory follicles in response to LH surge becomes essential. In this regard, expression of many genes in the dominant follicle identified to be down or up regulated in response to hCG injection or endogenous LH surge have been reported for cattle [14], [15]. However, the analysis of global changes in gene expression patterns in response to gonadotropin surge has not been reported. Several genes associated with the apoptotic signaling cascade were observed to be differentially regulated in the dominant versus subordinate follicles [16]. A functional Fas-mediated apoptotic pathway has been characterized in the bovine follicles and cell survival appears to be mediated mainly by E_2_ levels and IGF-I mediated activation of PI3K by modulating the progression of GCs through the cell cycle [17]–[20]. The expression of genes involved in ECM remodeling and follicular rupture viz., PLAT, PLAU, SERPINE 1 and 2, MMP-1, -2, -13 and -14, TIMP-1, -2, -3 and -4, ADAMTS subtypes and HPSE have been reported to be regulated in response to the gonadotropin surge [21]–[28].

Several *in vitro* studies have demonstrated the role of IGF-I in the regulation of cell proliferation, survival and steroidogenesis in GCs. E_2_ production appear to be regulated by intrafollicular concentrations of IGF-I and IGF-II [29], and concentrations of these may in turn be modulated by IGF binding proteins. IGF-I promotes proliferation of GCs as well as progesterone (P_4_) production by medium and large follicles with FSH having an additive effect [30], [31]. Whereas, IGF-I in the presence of LH stimulates thecal cell androgen and P_4_ production *in vitro*, which gets inhibited following addition of IGFBP2 [32], [33]. Levels of IGFBP2, 4 and 5 are low in the follicular fluid of the dominant follicle correlating with the presence of increased pregnancy associated plasma protein A (PAPPA) activity [34]–[37]. PAPPA mRNA expression and activity are stimulated by FSH and occurs concomitant with the increased follicular fluid E_2_ levels [37]. These and many other studies, have examined the role of IGF-I during the ovarian follicular growth and acquisition of dominance, however, the role of IGF-I during the periovulatory period remains to be delineated. To date, effects of bovine growth hormone (bGH) on circulating IGF-I levels as well as direct and/or indirect actions of bGH on the gonadal function have not been described in the buffalo cow. Moreover, the global gene expression data generated in this study suggest differential expression of many genes associated with IGF-I system and its signaling in the preovulatory follicle. In light of the above, the following objectives were examined: (i) Analysis of global gene expression changes in GCs of the ovulating follicle, (ii) effect of administration of exogenous bGH to transiently increase circulating and intrafollicular IGF-I concentrations during the periovulatory period, and (iii) studies on changes in expression of genes regulating steroidogenesis, cell survival and differentiation in response to increased IGF-I levels during the periovulatory period.

## Materials and Methods

### Reagents

Juramate® (Cloprostenol sodium, a synthetic analogue of PGF_2α_) was procured from Jurox Pty Ltd, Rutherford, Australia. DyNAzyme™ II DNA polymerase was purchased from Finnzymes, Espoo, Finland. MMuLV reverse transcriptase was obtained from MBI Fermentas GmbH, St. Leon-Rot, Germany. Power SYBR® Green PCR master mix was obtained from Applied Biosystems, Foster City, CA. Anti-bovine LH (USDA-309-684P), anti-bovine FSH (AFP-C5288113) antisera, iodination grade bovine LH (AFP-11743B) and FSH (AFP-5332B) were procured from Dr. A. F. Parlow, NHPP, Torrance, CA. bGH was a kind gift from Monsanto Company St. Louis, MO. IGF-I immunoradiometric assay DSL-2880 kit was from Diagnostic System Laboratories, Webster, TX. Antibodies specific to IGFBP2 (M-18; sc-6002), IGFBP3 (M19; sc-6004), and p27 (C-19; sc-528) were procured from Santa Cruz Biotechnology Inc., CA and Anti-FKHR (#9462) from Cell Signaling Technology, Beverly, MA. Oligonucleotide primers were synthesized by Sigma-Genosys, Bangalore, India. GnRH and all other reagents were purchased from Sigma Aldrich Corporation, Bangalore, India or sourced from local distributors.

### Experiment 1: Gene expression profiling of the preovulatory follicle immediately before and after occurrence of the gonadotropin surge

**Figure 1:**
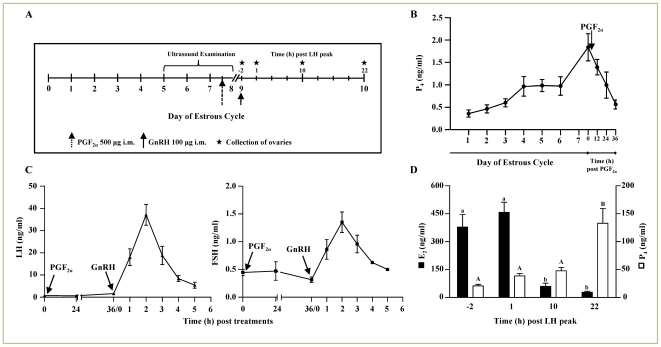
Standardization of experimental animal model for characterization of periovulatory events during the LH surge. (A) Schematic representation of the experimental protocol employed for collection of follicular contents before and at different time points after peak LH surge. Follicular fluid, granulosa cells and follicle wall of the ovulatory follicle were harvested from the ovaries collected at the indicated time points for carrying out various analyses. (B) Circulating concentration of P_4_ during different days of estrous cycle for collection of follicular contents before and at different time points post peak LH surge. Serum P_4_ level was determined in blood samples collected daily beginning on day 1 of estrous cycle and at 12 h intervals after administration of PGF_2α_ and GnRH treatments. For different days of estrous cycle, values from all animals recruited for this experiment (n = 23) were pooled and represented as mean ± SEM. (C) Mean ± SEM serum concentration of LH and FSH during the preovulatory period. (D) Follicular fluid concentration (mean ± SEM) of E_2_ and P_4_ at different time points before and after peak LH surge (n = >5 animals/time point). For each hormone, bars with different superscripts are significantly different (p<0.05).

Experiments (Experiment 1 and 2) in buffalo cows described in this study were cleared by the institutional animal ethics committee, Indian Institute of Science, Bangalore, India. The validation of experimental model system employed for studying gene expression profiling of the ovulating follicle has been provided as Data S1. The details of protocol employed in this experiment are represented in Figure 1A. Briefly, to monitor various hormone concentrations, blood samples were collected from the experimental non lactating buffalo cows. On day 7 of the cycle, Juramate (500 µg, i.m.) was administered to animals to induce luteal regression and initiation of follicular phase. GnRH (100 µg, i.m.) was administered 36 h post Juramate injection to induce gonadotropin surge. Serum samples were subjected to assay of P_4_ and gonadotropin concentration to verify CL regression and to determine the timing of LH surge. Ovaries were collected at slaughter before, 3, 10 and 24 h post GnRH injection (n = 4 animals or more/time point), following confirmation of time of peak LH surge, these time points correspond to −2, 1, 10 and 22 h post peak LH surge. These time points were chosen with a view to catalogue gene expression changes in the preovulatory follicle at the earliest time point post peak LH surge (i.e., 1 h) and at a later time point (i.e., 22 h) the time point closest to the time of ovulation. Ovaries containing preovulatory follicles were processed for retrieval of GCs and the follicle wall, as reported previously [38].

### Experiment 2: Effects of bGH on IGFBP expression and various hormone concentration

After having determined the gene expression profiling of GCs of the preovulatory follicle at different time points, the differentially expressed genes were further subjected to pathway analysis (Fig. S3). Further, to examine direct effects of various peptides/growth factors on responsiveness of the preovulatory follicle to the gonadotropin surge, different experimental models were explored and only studies on IGF-I experiments are discussed here. We tested the effects of increased milieu of IGF-I on gonadotropin actions in the preovulatory follicle. Due to practical difficulties that included the exorbitant cost involved in direct administration of exogenous IGF-I to the animals, we chose to stimulate the secretion of endogenous IGF-I by exogenous administration of bGH. To rule out that GCs of the preovulatory follicle are direct targets of bGH, we determined the GHR expression, which was undetectable in GCs from the follicle obtained under different experimental conditions, while GHR expression in liver tissue was clearly demonstrable (Fig. S4). We next carried out several pilot studies involving various doses, single and multiple injections of bGH to select a dose (40 mg/injection) suitable for eliciting significant and sustained increases in circulating IGF-I concentration during the preovulatory period. The experiments were carried out in non-lactating multiparous animals essentially identical to that described in experiment 1, except that bGH treatment was incorporated into the protocol beginning on day 7 of the estrous cycle. Animals received Juramate and 4 ml of vehicle (Veh; n = 8 animals, 0.9% NaCl, 0.025 M NaHCO_3_ and 0.025 M Na_2_CO_3_) or 40 mg of bGH (10 mg/ml) at 24 h intervals up to 72 h; n = 4 animals. At 36 h post Juramate injection, ovaries were collected from the Veh treated animals (n = 4) at slaughter for follicular fluid and GCs collection as reported earlier [38], the remaining animals receiving Veh and bGH were administered GnRH 100 µg i.m., and GCs were collected from the periovulatory follicle using ultrasound guided needle biopsy post treatment. These time points of GCs collection correspond to −2 h and 22 h post peak LH levels.

### RNA isolation

Total RNA was isolated from GCs, follicle wall and the liver tissue using TRI reagent followed by a cleaning step using the RNeasy mini kit for RNA purification. RNA samples with RNA integrity number >7 in the Agilent Bioanalyzer 2100 were subjected for microarray analysis, three Affymetrix GeneChip Bovine Genome Arrays were used/time point as biological replicates i.e., a total of nine arrays were used.

### Microarray target preparation and hybridization

Transcriptome analysis of GCs was performed on GeneChip Bovine Genome Array (Affymetrix, Santa Clara, CA) which contains 24,027 probe sets representing ∼23,000 bovine transcripts and few housekeeping/control genes. Use of heterologous array hybridization screening for gene expression changes of closely related species has been validated for many species [39]. Affymetrix Bovine GeneChip platform is very efficient and can be easily extended to other species for which genetic sequences are present in public databases [40], [41]. The water buffalo and domestic cattle, both belonging to Bovidae family are closely related and additionally, the use of robust multiarray average (RMA) algorithm for background correction, normalization across arrays and summarization is well suited for cross species microarray analysis (since the software considers hybridization from perfect match probes and not the mismatch probes) [40].

Since the quantity of total RNA obtained from GCs in each animal was less, a linear two step amplification method using protocols recommended by Affymetrix GeneChip® expression analysis technical manual was employed. Briefly, in the first cycle 100 ng of total RNA from each sample was used for single strand cDNA synthesis using GeneChip® Two-Cycle cDNA synthesis kit and T7-Oligo dT primer. Single strand cDNA was then converted into double stranded cDNA using E.coli DNA Polymerase I and further, cRNA was prepared by an *in vitro* transcription (IVT) using MEGAscript T7 kit. In the second cycle, this cRNA was used to synthesize single strand cDNA employing random hexamers as primers before the second strand cDNA was synthesized using E.coli DNA Polymerase I. After two cycles of linear amplification, the 12–15 µg of cDNA obtained was in vitro transcribed using GeneChip® IVT labeling kit to generate Biotin labeled cRNA. The quality of biotinylated cRNA synthesized was verified using Bioanalyzer and further, 10 µg of biotin labeled cRNA was fragmented and hybridized to the Bovine GeneChip® Array. The entire procedure of microarray target preparation, hybridization and scanning was performed at Center for Integrated Biosystems, Utah State University, Utah.

### Semi-quantitative RT-PCR analysis

Semi-quantitative RT-PCR analysis was carried out essentially as described previously [38]. The details of primers employed along with the annealing temperature and expected amplicon size are provided in Table S1.

### Real-time quantitative RT-PCR (qPCR) analysis

qPCR analysis was carried out essentially as described previously [42]. Expression of L19 was used as internal control for normalization of each gene studied. The list of genes and details of the primers employed in the qPCR analysis along with the annealing temperature and expected amplicon size are provided in Table S2.

### Hormone assays

E_2_ and P_4_ concentrations were determined by specific RIAs as reported previously [38]. Follicular fluid diluted (E_2_: 1∶500 and 1∶1000; P_4_: 1∶10 and 1∶100) with 0.1% gelatin–PBS was used for assays without ether extraction. The sensitivity for E_2_ and P_4_ in the assays was 39 pg/ml and 0.1 ng/ml, respectively, the inter- and intra-assay coefficients of variation were <10%.

### LH and FSH

The bLH and bFSH RIAs consist of anti-bovine LH/FSH antisera and iodination grade bLH/FSH used both for iodination and preparation of standards. The antibody was used at a final assay tube dilution of 1∶135,000 (bLH) or 1∶96,000 (bFSH) in a total assay set-up volume of 300 µl. The average sensitivity of the assay was 0.2 and 0.125 ng/ml for LH and FSH, respectively. For both hormones, the inter- and intra-assay coefficients of variation were <10%.

### IGF-I

Concentrations of IGF-I in plasma and follicular fluid were measured using a commercially available Human ELISA kit. The sensitivity of the assay was 1 pg/ml. The inter- and intra-assay coefficients of variation were 7.6 and 6.2%, respectively.

### Immunoblotting analysis

Immunoblot analysis was carried out as per previously published procedures [43].

### Immunocytochemistry analysis

Immunocytochemistry analysis was carried out as per previously published procedures [43].

### Statistical analysis

Data were expressed as mean ± SEM. Statistical evaluation of mean differences of serum steroid, gonadotropins, IGF-I concentrations and qPCR fold change in expression among different experimental groups were analyzed using one-way ANOVA followed by the Newman-Keuls multiple comparison tests (PRISM GraphPad; GraphPad Software Inc., San Diego, CA). Student t- test was employed to compare between two groups. A p value of <0.05 was considered statistically significant.

### Analysis of microarray data

A detailed description of microarray assay procedures and subsequent generation of processed image files has been reported previously [42]. See Data S2 for all information necessary for Minimum Information About Microarray Experiments (MIAME) - compliance. The raw data and the completed analysis of microarray data files discussed in this publication have been deposited at NCBI's Gene Expression Omnibus (http://www.ncbi.nlm.nih.gov/gds?term=GSE11312) and are accessible through GEO Series accession number GSE11312. The processed image files (.cel) were normalized across arrays using the robust multichip average (RMA) [44] algorithm and log-transformed (base 2), thus allowing direct comparison of probe set values between all samples used in the experiment normalization, GeneSifter (VizX Labs; Seattle, WA) microarray expression analysis software was used to identify differentially expressed transcripts. The differential expression of genes was calculated by averaging the normalized samples and performing a pairwise analysis. Statistical significance for differentially expressed genes among control and treatment groups was determined using Student t-test (two tail, unpaired) employing Benjamini and Hochberg correction factor for false discovery rate [45] and the analyzed data is provided in Data S3&S4. The top 15 differentially regulated genes at both 1 and 22 h post peak LH surge are represented in Tables S3, S4, S5, S6. The linear regression analysis was performed on log_2_-transformed expression ratios obtained from qPCR and microarray experiments and a statistically significant (p<0.05) correlation between the two analyses were determined as reported previously [42]. As many of the original annotations for the Affymetrix Bovine Genome Chip have been reported to be erroneous [46]. A computational tool, AffyProbeMiner, was employed to redefine the chip definition files taking into account the most recent genome sequence information [47]. AffyProbeMiner analysis results suggested that the original Affymetrix gene annotation were not compromised and the identified differentially expressed genes were transcript consistent and did not hybridize to multiple transcripts. Further, to examine the molecular function and genetic networks, the microarray data were analyzed using Ingenuity Pathways Analysis tool (IPA version 8.7, Ingenuity® Systems Inc., Redwood City, CA, USA; http://www.ingenuity.com), a web-based software application which enables identification of biological mechanisms, pathways, and functions most relevant to experimental datasets or differentially expressed genes.

## Results

### Validation of the experimental model, concentration of steroids and gonadotropins during the periovulatory period

Figure 1A illustrates the experimental protocol employed for timed gonadotropin surge, ovulation and for collection of follicular fluid, GCs and follicle wall from the ovulating follicle. Circulating serum P_4_ level from all animals utilized in the experiment on each day of estrous cycle before and after administration of PGF_2α_ injection is represented in Fig. 1B. Progesterone concentration declined significantly (p<0.05) to reach 0.56±0.1 ng/ml 36 h post PGF_2α_ treatment. Circulating LH concentration was low during PGF_2α_ treatment, but increased significantly (p<0.05) after GnRH administration to reach peak concentration of 37.2±4.7 ng/ml, 2 h post GnRH injection and declined thereafter to return to basal concentration within 5 h (Fig. 1C). Similar to LH, circulating FSH concentration was low prior to GnRH injection, increased significantly to peak at 2 h, and concentration declined thereafter (Fig. 1C).

### Biochemical analysis of the preovulatory follicle

Since ovaries containing preovulatory follicle were collected before ovulation, the physiological end point (ovulation) in response to hormone treatments could not be monitored, instead circulating P_4_ and LH levels were measured to verify that animals responded to treatments (Fig.1B & C). Follicular fluid steroid levels and expression of genes considered as marker of the ovulating follicle were monitored and the results are presented in Fig. 1D and Fig. S1(A–C), respectively. Follicular fluid E_2_ concentration from follicles collected 2 h before peak LH surge was 378.8±66.5 ng/ml and increased to 458.0±53.8 ng/ml within 1 h peak LH surge, whereas, it was lower in follicles collected at later time points. Follicular fluid P_4_ concentration from follicles collected before peak LH surge was low to start with and progressively increased to reach 133.0±26.2 ng/ml in follicles collected at 22 h post peak LH surge (p<0.05; Fig. 1D).

### Gonadotropin-induced gene expression changes in GCs of the preovulatory follicle

**Figure 2:**
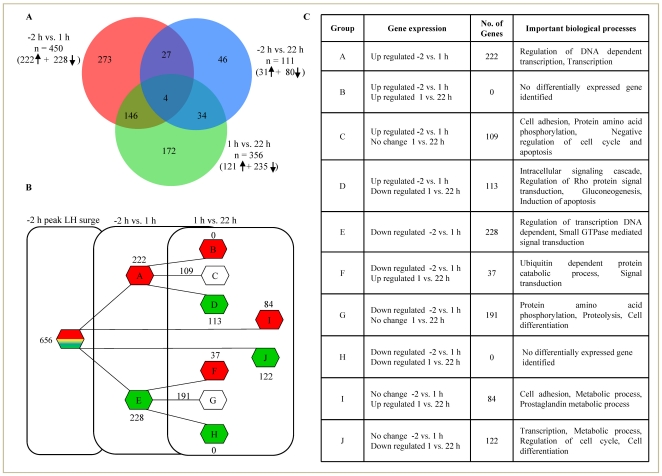
Schematic representation of temporal changes in differentially expressed genes at 1 h and 22 h post peak LH surge. (A) Venn diagram representing the details of differentially expressed genes identified after microarray analysis of granulosa cells collected from the ovulatory follicle at −2, 1 and 22 h post peak LH surge. Data analyzed with ≥2 fold change cut-off and statistics. The number of differentially expressed genes found common between −2 vs. 1 h (red circle) and −2 vs. 22 h (blue circle) post peak LH surge, as well as comparison of differentially expressed genes between 1 vs. 22 h (green circle) post peak LH surge are presented. (B) Schematic representation of differentially expressed genes at different time points post peak LH surge. Red hexagon represents genes that were up regulated both at 1 and 22 h as well as those genes that were down regulated at 1 h but were up regulated at 22 h time point. Green hexagon represents differentially expressed genes that were down regulated both at 1 and 22 h time points as well as those genes that were up regulated at 1 h but were down regulated at 22 h time point. Also provided is the number of genes (represented as open hexagon) that were differentially expressed at 1 h time point but not at 22 h. (C) The number of differentially expressed genes listed as alphabetical groups in Fig. 2B are further represented in tabular form providing details on the number of genes in each group, pattern of expression change at both time points post peak LH surge as well as classification based on their ontological distribution within each ‘biological processes’ terms having high scores indicative of processes associated with each group.

Microarray analysis results (GEO # GSE11312), after subjecting to high stringency statistical analysis revealed 450 genes to be differentially expressed within 1 h of peak LH surge (≥2 fold change with Benjamini and Hochberg correction for false discovery rate), of these 220 and 228 genes were up and down regulated, respectively. The analysis of microarray data at 22 h post peak LH surge revealed 111 genes to be differentially expressed, of which 31 genes were up regulated, while 80 genes were down regulated (Fig. 2). A Venn diagram in Fig. 2A describes the number of genes differentially up or down regulated within a time point examined and the number of differentially expressed genes found common between time points (−2 vs. 1 h and −2 vs. 22 h post peak LH surge). The Venn diagram also provides the comparison of differentially expressed genes between 1 and 22 h post peak LH surge time points. Further, distribution of nearly 656 differentially expressed genes from −2 to 1 and 22 h post peak LH surge time points is represented in pictorial and tabular form in Fig. 2B&C. Most cluster of genes were observed to be modulated in a synchronous way at the two temporal points post peak LH surge i.e., higher percentage of the genes regulated at 1 h time point had an opposite regulation at 22 h time point.

The top fifteen differentially expressed genes at time points 1 and 22 h post peak LH surge were determined and the data are represented as top up and down regulated genes in Tables S3, S4, S5, S6. Analysis of top up regulated genes at 1 h post peak LH surge indicated that the differentially expressed genes belonged to cellular signaling (PLK2, TNFAIP6, ATF3, CNKSR3, NR4A1, RGS16, ARHGEF3 and BAMBI) and matrix modulators (ADAMTS1&4 and CTGF). In the list of top down regulated genes, most of them belonged to cell cycle regulation and apoptosis (ASPN, PAWR and FOXOA1). At 22 h post peak LH surge, analysis of list of top up regulated genes indicated that most differentially expressed genes belonged to pericellular matrix proteoglycan family which include proteins like Claudins (CLDN11-tight junctions), Decorins (DCN) and lumicans (LUM) of small leucine-rich proteoglycan (SLRP) family; collagens (COL3A1 and COL1A1), procollagens, CTGF and SERPINE1 indicative of the transformation of granulosa cells in to luteal tissue architecture. Further, the list of top down regulated genes contained differentially expressed genes that belonged to coagulation system (ASPN, F2RL1 and LYZ), steroidogenesis (CYP19 and HSD17B1), Wnt and IGF signaling [SFRP1&3(FRZB), IGFBP4].

For validation of differentially expressed genes at the two time points examined, 20 genes belonging to different ontological categories were selected for qPCR analysis. The cumulative fold changes of differentially regulated genes (PAPPA, BCL-2, IGF-IR, IGFBP3, HSD17B1, CYP19, IGF-IIR, IGFBP2, PLAT, STAR, CTGF, PLAU, PIAS1, ASPN, protease activated receptor (PAR-2)/F2RL-1, Thrombomodulin (THBD), PAWR, FKHR/FOXOA1, FRZB1 and SERPINE-1) for both microarray and qPCR analyses are represented in Fig. S2A &B. Upon subjecting to goodness of fit analysis (log ratio comparison data provided in Fig. S2C), except for the expression of F2RL-I, THBD, PLAU and IGF-I R, IGF-II R, PLAT genes, at 1 h and 22 h time points, respectively, qPCR analysis for most genes corroborated well with the microarray data. The differential regulation of FKHR was also examined at the protein level by immunocytochemistry analysis (Fig. S2D). Expression of FKHR prior to the onset of peak LH surge was generally localized to the nucleus of GCs, and it decreased drastically at 22 h post peak LH surge [Fig. S2D (A–H)].

### Pathway analysis of microarray analyzed data

**Figure 3:**
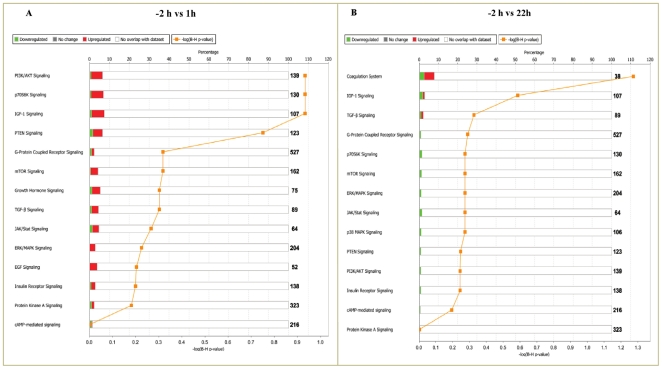
Classification of differentially expressed genes into various canonical pathways by Ingenuity pathway analysis (IPA). The pathway analysis indicates that a large number of differentially expressed genes belonged to canonical pathways such as PI3K/AKT signaling; IGF-I signaling and coagulation system. The orange line represents score for the likelihood [−log (B–H *P*<0.05)] that genes belonging to a specific signaling pathway category affected at 1 and 22 h post peak LH surge. The stacked bars indicate the percentage of genes distributed according to regulation i.e. green (down), red (up) and open bars (No overlap with dataset) in each canonical pathway.

**Figure 4:**
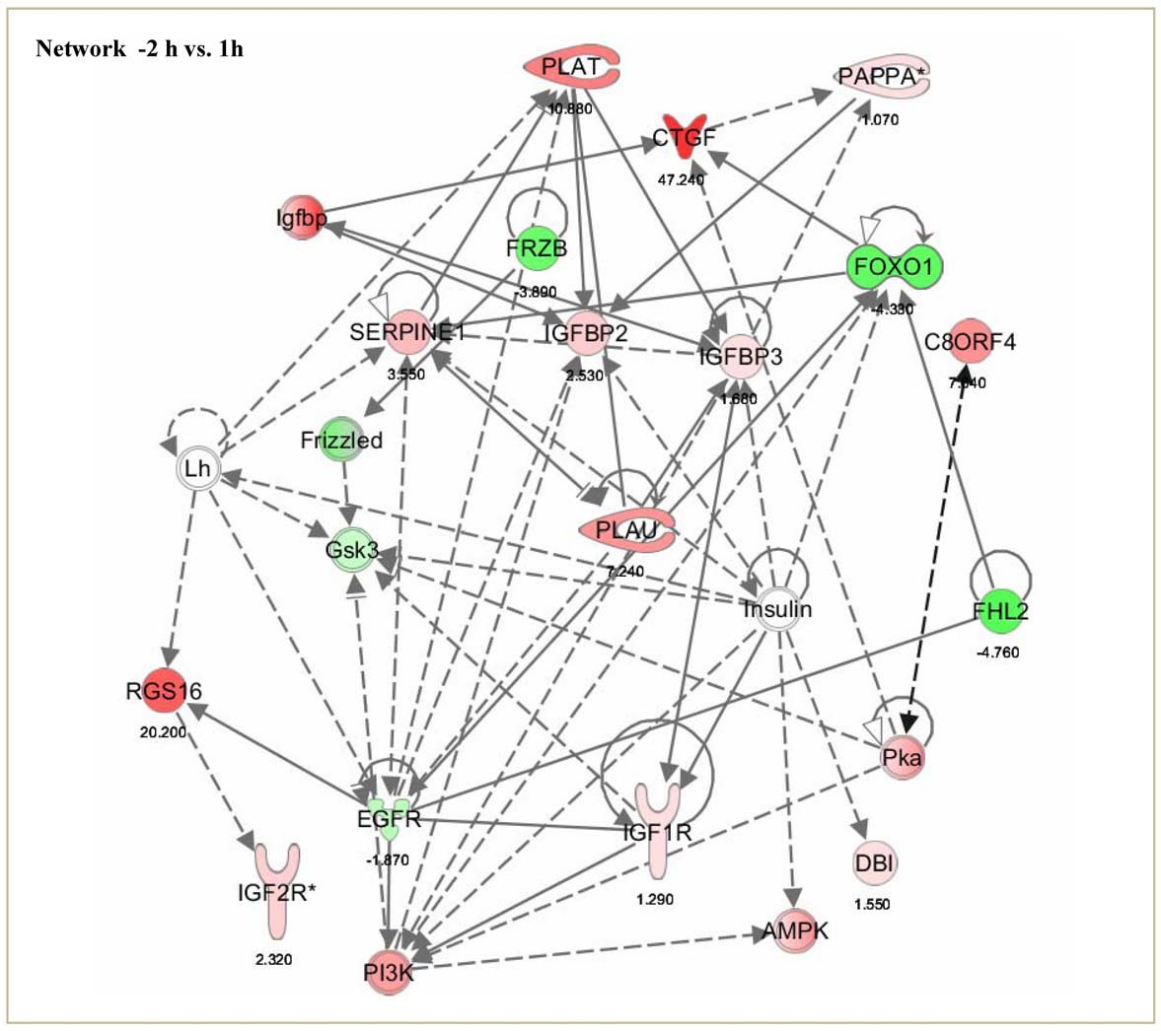
Network 1; Ingenuity pathway analysis of the differentially regulated genes at 1 h post peak LH surge shows a network of 22 focus molecules with a score of 40, with top biological functions of cell-to-cell signaling and interaction, cell cycle and tissue development and morphology. The network is displayed graphically as nodes (genes/gene products) and edges (biological relationship between nodes). The node colour intensity indicates the fold change expression of genes; with red representing up-regulation, and green down-regulation of genes between −2 vs. 1 h post peak LH surge in granulosa cells. The fold change value for individual gene is indicated under each node. The shapes of nodes indicate the functional class of the gene product and the lines indicate the type of interaction.

**Figure 5:**
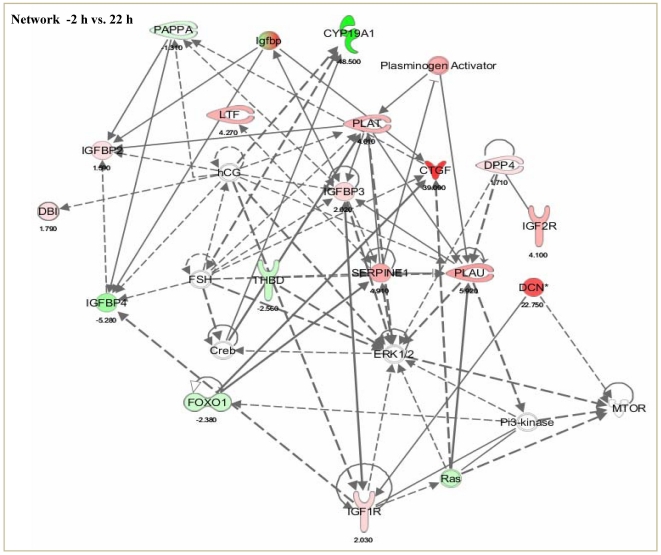
Network 2; Ingenuity pathway analysis of the differentially regulated gene at 22 h post peak LH surge shows a network of 20 focus molecules with a score of 35, with top biological functions of cell-to-cell signaling and interaction, cell death, and endocrine system disorders. The network is displayed graphically as nodes (genes/gene products) and edges (biological relationship between nodes). The node color intensity indicates the fold change expression of genes; with red representing up-regulation, and green down-regulation of genes between −2 vs. 22 h post peak LH surge in granulosa cells. The fold value for individual gene is indicated under each node. The shapes of nodes indicate the functional class of the gene product and the lines indicate the type of interaction.

Ingenuity pathway analysis (IPA) was used to classify the differentially expressed genes into different function and disease categories. Of the 450 and 111 differentially expressed genes at 1 and 22 h, respectively, 290/66 genes were determined to be network eligible of which 240/59 genes were determined to be function/pathway or list eligible. Under the list of biological category, the largest number of down regulated genes was associated to cell to cell signaling and interaction, cellular growth and proliferation. Gene classification according to canonical signaling pathways revealed that genes associated with IGF-I signaling, coagulation system and PI3K/AKT signaling were the categories containing largest number of genes differentially regulated post LH surge as per the IPA terminology (Fig. 3 and Fig. S5). Genes most significantly affected post peak LH surge, when classified into molecular and cellular functions revealed top 15 functions to be cell to cell signaling and proliferation related as shown in Fig. S3. The top few genes most dramatically up regulated with a fold change greater than 5.5 (p<0.05) at 1 h post peak LH surge were PLK2 (7.98), ADAMTS1 (5.69) and CTGF (5.56) genes. There was also increased expression of other genes involved in the regulation of cell to cell signaling and interaction. The top few genes which displayed the most dramatic down regulation were RASL11B (−3.52), ASPN (−2.73) and FKHR (−2.11). Whereas, the genes such as CTGF (5.29) and ASPN (−3.89) also had stronger regulation at 22 h post peak LH surge. From IPA, 35 networks (p<0.05) were identified with 20 networks each having 10 or more focus genes among the differentially expressed genes. At 1 h, the top gene network identified was cell-to-cell signaling and interaction, cell cycle and tissue development and morphology. (Network 1; score 40, 25 focus molecules; Fig. 4), while at 22 h the significant gene network identified was cell death, cell-to-cell signaling and interaction and endocrine system disorders. (Network 2; score 35, 20 focus molecules; Fig. 5).

### Gene expression patterns of the ovulating follicle

**Figure 6:**
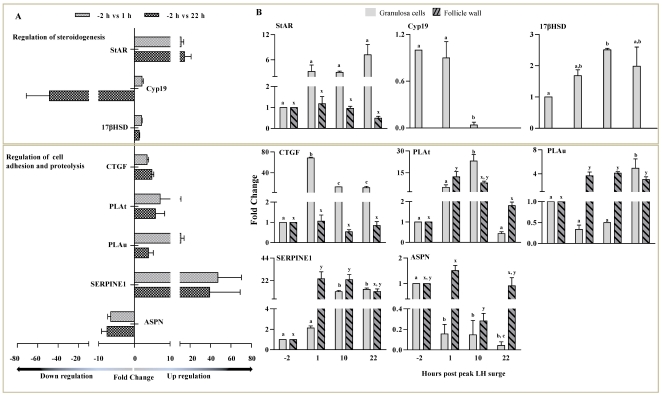
Expression of genes associated with steroidogenesis, cell adhesion and proteolysis in the periovulatory follicle. (A) Microarray fold expression changes at 1 and 22 h post peak LH surge in granulosa cells for few of the selected up and down regulated genes associated with specific biological process. (B) The genes selected by microarray analysis were also subjected to qPCR analysis and fold expression changes for granulosa cells and follicular wall collected at different time points are represented as bar graphs. Bar for each gene represents mean ± SEM fold expression change value at each time point (n = 3 animals). For each gene, bars with different alphabets above them are significantly different (p<0.05). More details on the analysis are provided in the results section.

Figure 6 shows qPCR fold change in expression pattern with corresponding microarray data of selected genes associated with regulation of steroidogenesis, cell adhesion and proteolysis. Expression of StAR was shown to be up regulated after the onset of peak LH surge by both microarray and qPCR analyses, while its expression in the follicle wall did not change significantly (Fig. 6A&B). Expression of other steroidogenic genes, CYP19A1 and HSD17β showed marked changes in GCs, while their expression was not detectable in the follicle wall (Fig. 6B). Expression of CTGF, a marker of differentiation was up regulated in GCs after the occurrence of peak LH surge, whereas, its expression in the follicle wall did not appear to be regulated (Fig. 6B). Expression of PLAT, an ECM modulator showed up regulation at both 1 and 22 h time points in GCs by microarray analysis, whereas was high only at 1 and 10 h, but was low at 22 h time point in qPCR expression analysis (Fig. 6A&B). The PLAT expression in the follicle wall samples was similar to the pattern in GCs seen after the occurrence of peak LH surge i.e., up regulated at 1 and 10 h, whereas, down regulated at 22 h (Fig. 6B). Expression of PLAU, another ECM modulator was up regulated at all time points post peak LH surge in GCs by microarray analysis, but the expression was up regulated only at 22 h by qPCR analysis (Fig. 6B). In the follicle wall, the expression of PLAU was up regulated after occurrence of peak LH surge (Fig. 6B). Expression of SERPINE-1, an inhibitor of PLAT and PLAU was up regulated in GCs and follicle wall by both microarray and qPCR (except 1 h) analyses, (p<0.05; Fig. 6B). Expression of ASPN became down regulated after occurrence of peak LH surge in GCs, while in the follicle wall the expression appeared to be down regulated only at 10 h time point post peak LH surge (p<0.05, Fig. 6B).

**Figure 7:**
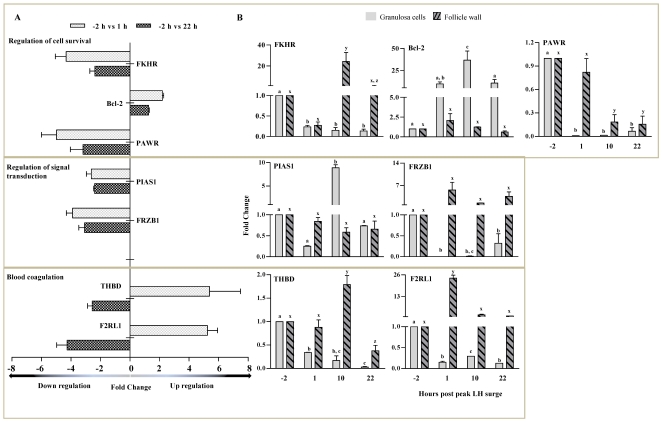
Expression of genes associated with cell survival, apoptosis and blood coagulation in periovulatory follicle. (A) Microarray fold expression changes at 1 and 22 h post peak LH surge in granulosa cells for few of the selected up and down regulated genes associated with specific biological process like cell survival, apoptosis and blood coagulation are represented as bar graph. (B) The genes selected by microarray analysis were also subjected to qPCR analysis and the fold expression changes for granulosa cells and follicular wall collected at different time points are represented in bar graphs. Bar for each gene represents the mean ± SEM fold expression change values at each time point (n = 3 animals). Bars with different alphabets above them are significantly different (p<0.05). More details on the analysis are provided in the results section.

Figure 7 shows qPCR fold change in expression pattern with corresponding microarray data of selected genes associated with cell survival, apoptosis and blood coagulation. Expression of BCL-2 gene was up regulated post peak LH surge with higher expression at 10 h time point. However, BCL-2 expression in the follicle wall did not change significantly (Fig. 7B). Expression of FKHR and PAWR in GCs were significantly down regulated after the onset of peak LH surge, while expression of PAWR was down regulated at 10 and 22 h time points and expression of FKHR was up regulated at 10 h in the follicle wall (Fig. 7B).

Expression of FRZB1 in GCs was down regulated at all time points examined, but in the follicle wall the expression did not change significantly. Expression of PIASI was low in GCs except for the 10 h time point and its expression did not appear to be regulated in the follicle wall post peak LH surge (Fig. 7B). Expression of F2RL-1 and THBD in GCs was up regulated at 1 h, whereas, down regulated at 22 h time point by microarray analysis, while expression of both genes was down regulated post peak LH surge (Fig. 7A&B). Expression of F2RL-1 in the follicle wall was low prior to the onset of peak LH surge, up regulated significantly (p<0.05) 1 h post peak LH surge, but was down regulated at 10 and 22 h time points. In the follicle wall, THBD expression was significantly up regulated at 10 h, but down regulated (p<0.05) at 22 h time point (Fig. 7B).

### Effects of bGH on IGFBP expression and various hormone concentrations

**Figure 8:**
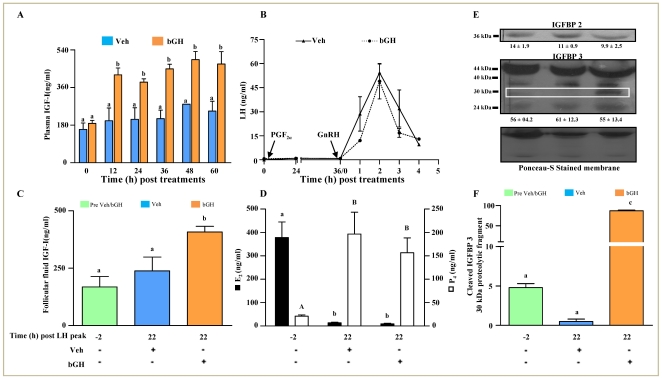
Effects of bGH treatment on IGFBP expression and various hormone concentration. (A) Circulating plasma concentration (mean ±SEM) of IGF-I in blood samples collected at different time intervals in Veh and bGH treated animals. Bars with different letters above them are significantly different (p<0.05). (B) Serum concentration of LH before and after GnRH administration to induce ovulation in buffalo cows treated with Veh (solid triangle) or bGH (solid circle). Concentration of LH was determined in blood samples collected at various time points before and after administration of GnRH to elicit endogenous LH surge. (C) Concentration of IGF-I (ng/ml) measured in the follicular fluid collected from follicles before (−2 h) and 22 h post peak LH surge of Veh or bGH treated animals. Bars with different letters above them are significantly different (p<0.05). (D) Mean ± SEM concentrations of E_2_ and P_4_ in follicular fluid collected from follicles before and at 22 h post peak LH surge of Veh or bGH treated animals. Bars with different alphabets within each hormone indicate significantly different concentration (p<0.05). (E) Protein expression of IGFBP2, IGFBP3 and Cleaved IGFBP3 in the ovarian follicular fluid. Protein lysates (100 µg) prepared from follicular fluid collected from the preovulatory follicle before (−2 h) and 22 h post peak LH surge of Veh or bGH treated animals were resolved on 10% SDS PAGE, transferred onto PVDF membrane and immunoblot analysis was performed using anti-IGFBP2 or anti-IGFBP3 antibody. The positions of 36 kDa IGFBP2 and 44–40 kDa IGFBP3 doublet bands are indicated by arrows and 30 kDa bands of IGFBP3 proteolytic fragment is highlighted in the boxed area. Densitometric values were determined and are indicated as mean ± SEM below the bands. The image of Ponceau-S stained membrane is depicted to indicate that equal amount of protein was loaded in each lane. (F) Densitometric analysis of immunoblots for 30 kDa IGFBP3 proteolytic fragment: mean ± SEM of relative amount of 30 kDa IGFBP3 proteolytic fragment expressed as intensity of Ponceau-S stained bands in each group (n = 3 animals/group). Bars with different letters are significantly different (p<0.05).

The data on expressions of PR and COX-2, regarded as marker of the ovulating follicle, is presented in Fig. S1D&E. Circulating plasma level of IGF-I in Veh and bGH treated animals is presented in Fig. 8A. A significant (p<0.05) increase in IGF-I level was observed within 12 h of bGH injection and the higher level was sustained throughout the observation period. The occurrence of endogenous surge of LH after sequential treatment with PGF_2α_ and GnRH was essentially identical between the two groups of animals (Fig. 8B). The IGF-I concentration in follicular fluid prior to peak LH surge was 168.4±45.4 ng/ml and increased to 238.5±59.8 ng/ml in Veh treated animals, while concentration increased significantly (p<0.05) to 407.9±24.5 ng/ml in bGH treated animals (Fig. 8C). The follicular fluid E_2_ and P_4_ concentrations were similar between two groups except that the P_4_ concentration at 22 h time point was marginally lower in the bGH treated animals (Fig. 8D).

Since, bioactivity of IGF-I depends on the ratio of its interaction with binding proteins (higher the ratio, greater the availability of biologically active form), expression of IGFBP2 and IGFBP3 proteins was determined in the follicular fluid. At 22 h time point IGFBP2 protein was significantly (p<0.05) lower in bGH treated animals (Fig. 8E). Of the different proteolytic fragments of IGFBP3 that could be detected, amount of 44 kDa fragment was not significantly different between Veh and bGH treated animals, however, expression of 30 kDa protein fragment i.e., the cleaved proteolytic fragment, was several fold higher in the follicular fluid of bGH treated animals suggestive of higher degraded fragment and thus lowered binding of IGFBP3 to IGF-I (Fig. 8F).

**Figure 9:**
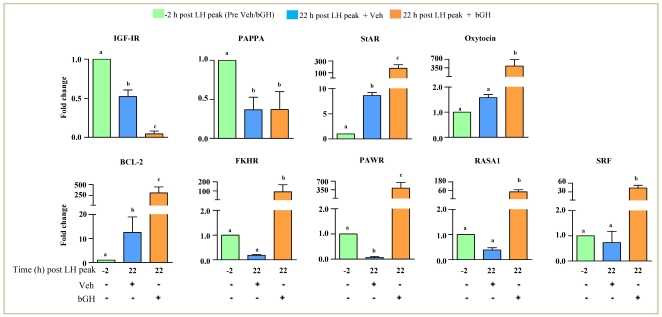
Gene expression changes in the preovulatory follicle of animals receiving bGH treatment. The qPCR fold change in expression pattern of selected genes associated with of IGF-I signaling (IGF-IR and PAPPA), luteinization (Oxytocin and StAR), cell survival, proliferation and differentiation (BCL-2, FKHR, PAWR, RASA1 and SRF) in granulosa cells of follicles retrieved before (green) and 22 h post peak LH surge of Veh (blue) or bGH (orange) treated animals. Bars with different alphabets above them are significantly different (p<0.05).

After having established the elevated IGF-I levels both in circulation and in follicular fluid, and possibly presence of more biologically active form, effects of IGF-I on expression changes of few of the genes associated with IGF-I signaling, luteinization process, tissue remodeling as well as apoptosis were examined and the results were presented in Fig. 9. Expression of IGF-IR in GCs of bGH treated animals was significantly lower at 22 h post peak LH surge (Fig. 9). Expression of PAPPA was low at 22 h post peak LH surge in GCs of both Veh and bGH treated animals. Expression of StAR and oxytocin, regarded as marker of luteinization and ovulation increased several fold at 22 h post peak LH surge, and the expression was significantly (p<0.05) higher in GCs of bGH treated animals (Fig. 9). Expression of transcription factors, FKHR and SRF, was significantly (p<0.05) higher in GCs of bGH treated animals at 22 h post peak LH surge (Fig. 9). Expression of BCl-2, PAWR and RASA-1 genes associated with apoptotic processes showed significantly higher expression in bGH treated animals compared to Veh treated animals (p<0.05; Fig. 9).

## Discussion

During spontaneous estrous cycles, the onset of preovulatory LH surge in buffalo cows is not entrained to a specific day of the cycle, consequently it is difficult to predict the time of onset of LH surge. The experimental model employed for studies in the buffalo cow is very much similar to the model system reported for cattle previously [48] and recently utilizing this model system, expression of up regulated genes has been reported for the cattle [15]. Since LH concentration peak at 2 h post GnRH administration both in cattle [48], [49] and buffalo cows (this study), it enabled us to fix the time point of GCs and follicle wall collection immediately before and at different time intervals post peak LH surge to examine events that unfold during early and at around the time of ovulation. In previous studies, others [2], [15] have observed less number of differentially expressed genes employing suppression subtractive hybridization or cDNA microarray techniques after hCG or GnRH treatments in cattle. In the present study, microarray analysis provided novel information on differential expressed genes within 1 h as well as at 22 h post peak LH surge which belong to cluster of genes associated with steroidogenesis, cell differentiation and survival and the expression of these genes have major bearing on initiation of ovulation and luteinization processes. The observation in our study that many of the identified genes were down regulated suggests an important role for LH in switching of expression of various genes associated with proliferation and metabolism. However, it should be pointed out that for further delineating a clear cut role of the various differentially expressed genes involved during different stages of ovulation process, the transcriptome analysis alone is insufficient, as the translated products of these genes ultimately provide a signature function. In the present study, except for qualitative determination of protein expression for two (for one protein data shown in Fig. S2) of the differentially expressed genes presented as immunocytochemistry data, the expression pattern for proteins of other differentially expressed genes was not determined due to limited availability of granulosa cells from the ovulating follicle for preparation of sample for immunocytochemistry and/or immunoblotting. More importantly, protein analysis is also hampered by lack of availability of specific antibodies that can detect proteins specific to the buffalo cow samples.

Analysis of the ontology report for the biological process category shows that initially in response to LH surge genes regulating cell survival, apoptosis and transcription were identified to be differentially regulated in the follicle, while at later time points, differential expression of genes involved in signal transduction, proteolysis, developmental process including vasculature development and wound healing was observed. It has been observed that bovine GCs gain resistance to apoptosis upon exposure to LH surge and this appears to be due to regulation of expression of pro-and anti- apoptotic factors via P_4_ signaling [50]. In the present study, expression of BCL-2 was up regulated in response to LH surge consistent with previous reports, in which gonadotropins reported to increase BCL-2 expression via PKA signaling [51]. It has been reported that PAWR, a pro-apoptotic gene regulates apoptosis in rat follicles and FSH acts by suppressing PAWR expression in the ovary by activating PKCζ-dependent anti-apoptotic pathway [52]. In the present study, PAWR expression was down regulated post peak LH surge suggesting activation of anti-apoptotic pathways. Pattern of down regulation of FKHR expression in response to LH surge and changes in its distribution from nucleus to cytoplasm post peak LH surge were similar to findings reported in rodents [53]. The function of FKHR during the periovulatory period depends not only on the transcriptional activity of the FKHR protein but also on the cellular milieu of pro- and anti-apoptotic factors as well as steroid hormone milieu, since FKHR is known to interact with nuclear steroid hormone receptors [54]–[56].

Extensive studies in bovines and primates have delineated important role played by VEGF, Angiopoietin (ANGPT) and FGF-2 during angiogenesis in preovulatory follicles and during the formation of CL [57]. In the present study, expression of some genes involved in angiogenesis, F2RL-1 and THBD was examined. F2RL-1 signaling mediates endothelial cell mitogenesis *in vitro*, and promotes vasodilatation and microvascular permeability *in vivo*, regarded as essential steps of the angiogenesis program [58]–[60]. The observation that, F2RL-1 expression was higher in the follicle wall samples may be important, since blood vessels from theca interna grow and invade luteinized mural GCs after follicular rupture. THBD is an endothelial cell surface glycoprotein that forms complex with thrombin and converts it as physiological anticoagulant. The down regulated expression of THBD in response to LH surge in the follicle wall indicates importance of physiological induction of coagulation during ovulation process [61]–[63]. CTGF, a cysteine rich peptide with strong angiogenic and tissue/ECM remodeling activities, has been shown to integrate processes of neo-angiogenesis and tissue remodeling both of which are necessary for formation of new CL [64]. An increased expression of CTGF observed in response to the LH surge suggests its involvement in angiogenesis and tissue remodeling processes. Another gene recently identified for its role in the regulation of ECM is Asporin, a novel member of leucine rich repeat family of proteins [65], [66]. Asporin is known to regulate the activities of cell surface receptors like TGFβ receptor and BMPR-IB thus preventing the activities of TGFβ and BMP-2 [67], [68]. In GCs, the role of down regulated expression of Asporin observed during LH surge remains to be determined.

In the present study, expression of various components of plasminogen activator system necessary for tissue remodeling and degradation of ECM were examined. In addition to its action on the ECM, plasmin activated by specific plasminogen activators, PLAT and PLAU has also been reported to activate another important protease system, family of matrixmetalloproteinases [69]–[72]. Increased expression of PLAT and PLAU observed in this study are similar to the findings reported in cattle [24], [73]. In the present study, the activities of plasmin, PLAT and PLAU were not examined, but it has been reported that their activities increase in the follicular fluid 12 h post gonadotropin surge, however, the PLAT activity at the base of the follicle was reported to be transient [24]. The up regulated expression of SERPINE-1 in response to LH surge and the pattern of its expression in GCs and follicle wall were in agreement with previous reports [23], [73].

Apart from the classical signal transduction pathways activated by gonadotropins and growth factors that have been investigated extensively, other pathways regulating the periovulatory events include Wnt, JAK-STAT, EGF and TGF signaling pathways [74]–[77]. Wnt 4 is expressed during follicular growth and is up regulated in response to LH surge, thus Wnt 4 is suggested to be involved in the regulation of target gene expression involved in ovulation [78]–[80]. The actions of Wnts are modulated by their antagonists, the secreted frizzled-related proteins. In the present study, expression of Frizzled B (FRZB), a secreted frizzled-related protein, was investigated. In response to gonadotropin surge, a drastic decline in the expression of Frizzled B was observed implicating possible role of Wnt signaling and its role in the activation of Wnt signaling during the periovulatory period in bovines. Recently, the Wnt/β-catenin signaling pathway during antral follicle development, ovulation and luteinization employing β-catenin gain of function and loss of function mouse models has been reported [78]. LH is also known to activate JAK-STAT pathway in GCs, in the present study, expression of PIAS1, an inhibitor of JAK-STAT signaling pathway, was down regulated in response to LH surge, except for a transient increase at 10 h time point, suggesting a role for JAK-STAT signaling during ovulation.

Another signaling system, whose role has been well documented during follicular development, but not during the periovulatory period, is the IGF-I signaling pathway. It was observed by Funston *et al.* [81] that IGFBPs other than IGFBP3 were not detectable in bovine preovulatory follicles and this may allow for increased availability of IGF-I required for oocyte maturation and ovulation. Although many of the actions of IGF-I have been reported on follicles, whether IGF-I regulates the maturation of oocyte cumulus expansion, luteinization or follicular rupture *in vivo* is not extensively investigated. In bovine and porcine species, *in vitro* studies employing GC and thecal cells have suggested that IGF-I regulates oxytocin and P_4_ production [82], [83] and has been shown to influence oocyte maturation and cumulus expansion [31], [84]–[88]. Intrafollicular injection of IGF-I has been suggested to effect increase or no change in E_2_ concentration in the follicles [89]. Because the follicle is permeable to small peptides, follicular fluid provides an index of integrated plasma proteins over time. Assay of IGF-I in follicular fluid of bGH treated animals revealed that the concentration paralleled increased circulating IGF-I concentration suggesting that increased levels may be responsible for most of the effects observed in GCs, however, whether exogenously administrated bGH also had direct effect remains to be investigated. Conflicting reports exist for the presence of GHR in bovine GCs, our findings of lack of expression of GHR in GCs of ovulating follicle confirm the finding by others [90], [91], but is in disagreement with some others [92]. However, absence of GHR expression in the theca was in agreement with the previous study [92]. The concentration of total IGF-I in the follicular fluid of the dominant and preovulatory follicle observed in the present study are in agreement with previous reports [81], [93]–[95] except for a report by Echternkamp *et al.* [96] who observed lower levels of IGF-I. This study demonstrated for the first time that increase in circulating IGF-I causes a significant rise in intrafollicular IGF-I levels implicating the importance of IGF-I from endocrine origin during the follicular growth. Several previous reports, have examined the binding activity of IGFBPs by ligand blotting assay [36], [37], [81], [93], [96], [97]. Immunoblotting analysis was carried out in the present study for examining the relative amounts of IGFBPs present in the follicular fluid and presence of both intact IGFBP2 and 3 were detected in the preovulatory follicle at all time points examined. Funston *et al* [77]. determined the levels of IGFBPs by ligand blot assay and only IGFBP3 was detected in the follicular fluid and this suggests that even though presence of IGFBP2 was detected in the present study, but has little capacity to bind IGF-I. Also, it was observed that amount of 30 kDa proteolytic fragment of IGFBP3 increased in bGH treated animals perhaps suggestive of activation of IGFBP3 specific protease such as PAPPA. The increased amount of 30 KDa proteolytic fragment of IGFBP3 in the follicular fluid suggests an increased activity of PAPPA however, involvement of other proteases such as MMPs and Cathepsin L cannot be ruled out [98]–[100].

Expression of some of the candidate genes involved in the regulation of steroidogenesis, cell survival, proliferation and differentiation as downstream targets of IGF-I action was examined. In the mouse Leydig cells, the transcription of StAR is known to be up regulated by IGF-I [101], [102]. In the present study expression of StAR was dramatically up regulated both in GCs and follicle wall following bGH treatment. In GCs, IGF-I in synergism with gonadotropins has been shown to increase transcription of StAR [103], [104]. Recent studies in porcine GCs suggest involvement of Kruppel-like factors in the increased transcription of StAR in GCs in the presence of LH and IGF-I [105]. In the present study, StAR protein expression in the follicular cells was not investigated due to lack of availability of sufficient number of cells. Surprisingly, even though StAR mRNA expression increased in response to increased intrafollicular IGF-I, no concomitant increase in follicular fluid P_4_ level was seen, instead the levels tended to be lower. It is debatable whether an increased transcription of StAR should lead to a rapid increase in P_4_ biosynthesis during initial stages of formation of CL, however, additional studies are required to confirm IGF-I's effect on luteal P_4_ production. An increased expression of oxytocin was observed in cells of bGH treated animals which is in agreement with previous *in vitro* GC studies in which the expression of oxytocin was increased in the presence of IGF-I [82], [106]–[108].

IGF-I is known to regulate cell cycle progression, proliferation and survival in bovine GCs [11], [19]. The increased expression of BCL-2 in GCs and follicle wall as observed in the present study is similar to the findings reported in melanoma and neuronal cells [109], [110]. However, it was observed that expression of pro apoptotic markers viz., FKHR and PAWR was up regulated in GCs. An important point to be noted here is that the means by which IGF-I and the LH surge confer resistance to apoptosis and promote cell survival are different. IGF-I mediates survival signaling by aiding the progression of cell cycle from G1 to S phase, where as LH surge promotes exit of GCs from the cell cycle and thereby conferring resistance to apoptosis [11], [19]. The increased intrafollicular IGF-I levels may have prevented the exit of GCs from the cell cycle due to increased expression of FKHR. Indeed, elevated expression of FKHR and its phosphorylation mediated by FSH and IGF-I relieves repression on cyclin D2 to promote proliferation, while decreased expression of FKHR and its localization to cytoplasm observed in response to LH surge appear to be associated with decreased proliferation [53], [111], [112]. Increased expression of PAWR in GCs indicates the increased susceptibility of GCs to undergo apoptosis. However, it has to be noted that there exists balance between pro- and anti- apoptotic factors that determines cell survival. RASA1 is a GTPase activating protein which increases the weak intrinsic GTPase activity of RAS proteins involved in the control of cellular proliferation and differentiation [113]. Studies on mutations in RASA1 have suggested that it plays an important role in the neoangiogenesis [114]. The expression of RASA1 was down regulated in response to LH surge, but GCs from bGH treated animals had higher expression of RASA1. The transcription factor, SRF activates multiple signaling pathways downstream of IGF-I [115]. The increased expression of SRF in bGH treated animals suggests modulation of differentiation process initiated by the LH surge.

In summary, the objectives of this research were to describe global gene expression changes in the ovulating follicle, and to determine whether transiently elevated IGF-I could influence the LH-mediated gene expression changes in the preovulatory follicle. Our findings describe rapid gene expression changes during LH surge and further suggest that expression of genes could be influenced by elevated intrafollicular IGF-I concentrations. The findings that elevated intrafollicular IGF-I level altered gene expression changes in the ovulating follicle suggest that gonadotropin actions during periovulatory period can be altered by growth factors that may change the outcome of process of ovulation.

## Supporting Information

Figure S1 **Characterization of expression of genes regarded as markers of the ovulating follicle.** Semi-quantitative RT-PCR analyses was carried out to examine the expressions of LHR (A) PR (B&D) and COX-2 (C&E) in granulosa cells collected from ovaries −2, 1, 10 and 22 h post peak LH surge in Veh (A–C) and bGH treated (D&E) animals. Total RNA 500 ng isolated from granulosa cells was reverse transcribed and cDNA equivalent of 25 ng of total RNA was used for PCR reactions. The data for semi-quantitative RT-PCR is represented as fold change over control (−2 h) for LHR, PR and relative expression levels for COX-2 after normalizing the band intensities of each PCR product with that of L19 for its corresponding sample. Each bar represents Mean ± SEM values for each time point (n = 3 animals/time point).
(TIFF)

Figure S2 **Validation of microarray data performed using GeneSifter analysis tool and its comparison with qPCR analysis.** (A, B) Comparison of gene expressions fold changes between microarray and qPCR analyses for selected genes in granulosa cells collected from the ovulating follicle. Mean ± SEM fold expression changes −2 vs. 1 h (A) and −2 vs. 22 h (B) for selected genes are represented in the bar diagram. (C) Correlation analysis between log ratios of expressions obtained from microarray and real-time qPCR analyses. Linear regression analysis was performed for selected differentially expressed genes for −2 vs. 1 h (left panel), −2 vs. 22 h (right panel); using log_10_-transformed qPCR relative expression values (2^−ΔΔCT^; Y-axis) and log_10_-transformed fold change expression values obtained by microarray analysis (X-axis). P value indicates the significance of the correlation as determined by F test. R, correlation coefficient generated for the theoretical line of best fit (represented as solid line in each panel). (D) Immunolocalization of FKHR in granulosa cells from follicles in response to gonadotropin surge −2 and at 22 h post LH peak surge. Immunolocalization was performed using antibody specific to FKHR (A–H), after probing signal visualized using FITC-conjugated ant-rabbit IgG. Sections were counterstained with Hoechst to localize nuclei. Data is representative of three independent experiments. Pictures shown in panels A–C and E–G are of the same magnification (63×) and that of panels D and H are enlarged to higher zoom. Bar: 5 µm.
(TIFF)

Figure S3 **Ingenuity pathway analysis of differentially expressed genes into various ontological groups.** Ingenuity pathway analysis classification of differentially expressed genes based on top 14 molecular and cellular functions, most significantly affected post peak LH surge in the granulosa cells. The blue bars indicate the likelihood [−log (B–H *p*-value)] that the specific ontological classification category was affected 1 (A) and 22 h (B) post peak LH surge compared with −2 h prior to peak LH surge. The threshold cut-off (orange line) is shown at *p*<0.05 (1.301 log scale).
(TIFF)

Figure S4 **Expression of GHR mRNA in liver and granulosa cells of buffalo cows.** Semi-quantitative RT-PCR analysis of GHR was performed in GCs collected from buffalo cows before and at 24 h post GnRH administration in animals treated with or without bGH. Total RNA (500 ng) isolated from liver and GCs was reverse transcribed and cDNA equivalent of 25 ng of total RNA was used for PCR reactions. The housekeeping gene, L19, was used as internal control. Shown here is a representative gel picture of GHR and L-19 PCR amplification products.
(TIFF)

Figure S5 **IGF-1 signaling pathway generated by Ingenuity pathway analysis software.** The two canonical pathways, PI3K/AKT signaling and IGF-1 signaling pathways were the major biological functions associated with genes differentially expressed post gonadotropin surge. Each node represents a protein and the shape of node indicates functional class that may be up regulated (red) or down regulated (green) in the differentially expressed genes dataset, while proteins in open nodes were not found to be regulated in the microarray dataset.
(TIFF)

Data S1 **Validation of the experimental model.** The results of validation of experimental model system employed for studying gene expression profiling of the ovulating follicle has been provided.
(DOC)

Data S2 **Information necessary for MIAME compliance.** The data provides the Minimum Information About a Microarray Experiment that is required to enable the interpretation of the results of the experiment unambiguously and potentially to reproduce the experiment.
(PDF)

Data S3 **List of differentially expressed genes at −2 vs. 1 h time points generated post microarray data analysis.** The differential expression of genes was calculated by averaging the normalized samples and performing a pairwise analysis. Statistical significance for differentially expressed genes among control and treatment groups was determined using Student t-test (two tail, unpaired) employing filter of ≥2-fold change with Benjamini and Hochberg correction factor for false discovery rate.
(XLS)

Data S4 **List of differentially expressed genes at −2 vs. 22 h time points generated post microarray data analysis.** The differential expression of genes was calculated by averaging the normalized samples and performing a pairwise analysis. Statistical significance for differentially expressed genes among control and treatment groups was determined using Student t-test (two tail, unpaired) employing filter of ≥2-fold change with Benjamini and Hochberg correction factor for false discovery rate.
(XLS)

Table S1 **List of primers employed for semi-quantitative RT-PCR analysis.** The details of primers employed for Semi-quantitative RT-PCR analysis along with the annealing temperature and expected amplicon size are provided.
(TIFF)

Table S2 **List of primers employed for qPCR analysis.** The list of genes and details of the primers employed in the qPCR analysis along with the annealing temperature and expected amplicon size are provided.
(TIFF)

Table S3 **List of top 15 UP regulated genes at −2 h vs 1 h post peak LH surge.** Microarray data analysis was carried out to obtain a set of differentially expressed genes based on statistics, a t-test with p<0.05 and multiple hypothesis testing (Benjamini and Hochberg comparison test) to reduce the false positives. The identified differentially expressed genes that passed the statistical filters were further selected based on their presence in 80% of the samples examined and then another fold change cut-off filter use employed to narrow down the list. Microarray analysis was preformed with ≥2 fold change as cut-off for identification of differentially expressed genes. Whereas, the top 15 differentially UP regulated genes at 1 h post peak LH surge are represented in this table. Probe Set ID: The identifier that refers to a set of probe pairs selected to represent expressed sequences on an array; Fold Change: It is a number describing changes in expression level of a gene compared between control and treatment; Regulation: The expression of a particular gene in treated sample compared to control sample; Gene ID: gene symbols extracted from Entrez Gene or UniGene; Gene Title: gene name extracted from Entrez Gene or UniGene. The genes validated and analyzed further in the study are highlighted in red and others are discussed in the results section.
(TIFF)

Table S4 **List of top 15 DOWN regulated genes at −2 h vs 1 h post peak LH surge.** The top 15 differentially DOWN regulated genes at 1 h post peak LH surge are represented. The list of genes validated and analyzed further in the study is highlighted in green, while the remaining genes are discussed in the results section.
(TIFF)

Table S5 **List of top 15 UP regulated genes at −2 h vs 22 h post peak LH surge.** The top 15 differentially UP regulated genes at 22 h post peak LH surge are represented. The list of genes validated and analyzed further in the study is highlighted in red, while the remaining genes are discussed in the results section.
(TIFF)

Table S6 **List of top 15 DOWN regulated genes at −2 h vs 22 h post peak LH surge.** The top 15 differentially DOWN regulated genes at 22 h post peak LH surge are represented. The list of genes validated and analyzed further in the study is highlighted in green and the remaining genes are discussed in the results section.
(TIFF)
