# Supplementary figures and images for: Gene Expression Profiling of Preovulatory Follicle in the Buffalo Cow: Effects of Increased IGF-I Concentration on Periovulatory Events
Source: PLoS One. 2011 Jun 20;6(6):e20754. doi: 10.1371/journal.pone.0020754 (PMC3119055; doi:10.1371/journal.pone.0020754)

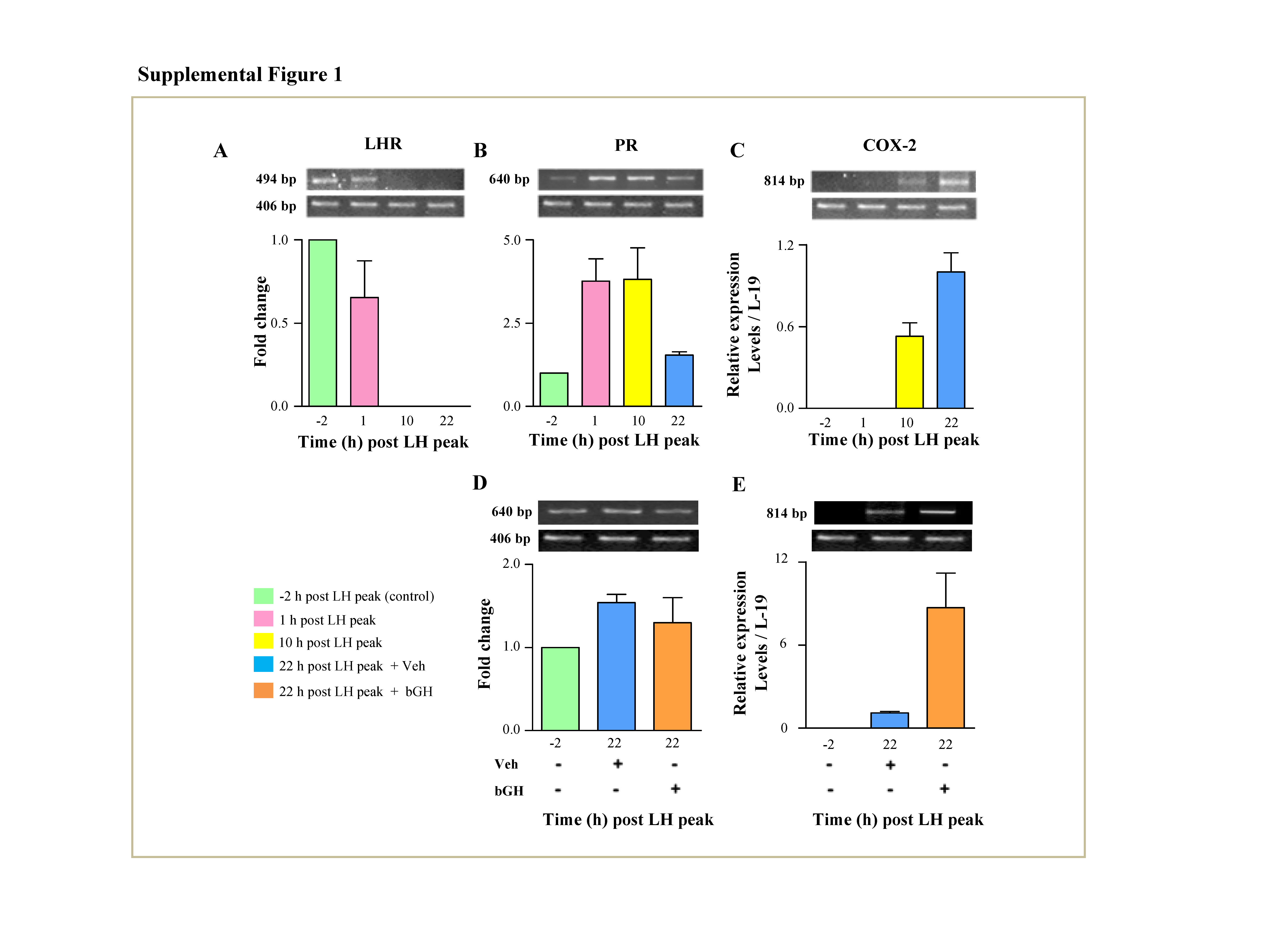

Supplement: Figure S1 — Characterization of expression of genes regarded as markers of the ovulating follicle. Semi-quantitative RT-PCR analyses was carried out to examine the expressions of LHR (A) PR (B&D) and COX-2 (C&E) in granulosa cells collected from ovaries −2, 1, 10 and 22 h post peak LH surge in Veh (A–C) and bGH treated (D&E) animals. Total RNA 500 ng isolated from granulosa cells was reverse transcribed and cDNA equivalent of 25 ng of total RNA was used for PCR reactions. The data for semi-quantitative RT-PCR is represented as fold change over control (−2 h) for LHR, PR and relative expression levels for COX-2 after normalizing the band intensities of each PCR product with that of L19 for its corresponding sample. Each bar represents Mean ± SEM values for each time point (n = 3 animals/time point). (TIFF) [file pone.0020754.s001.tiff]

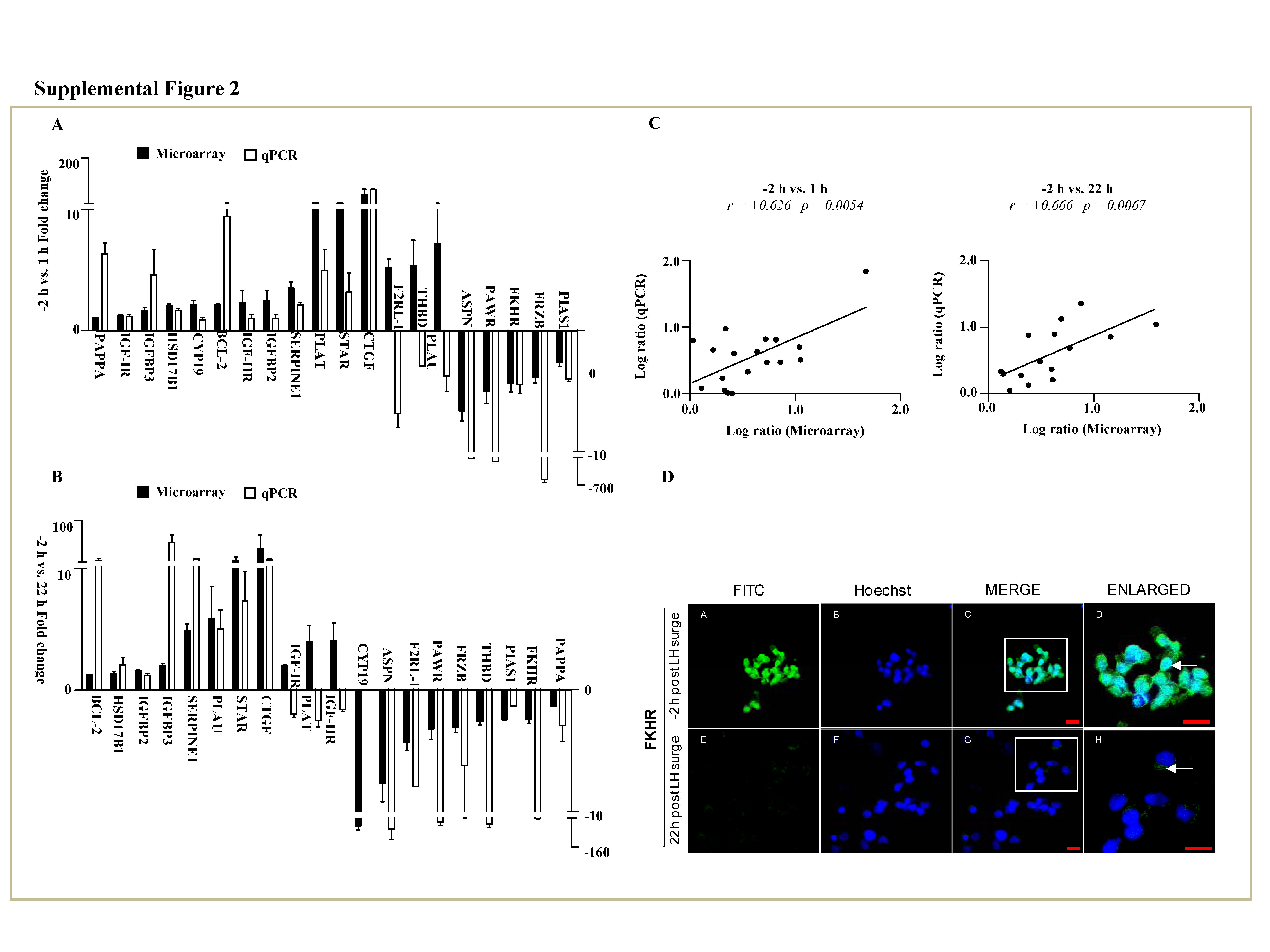

Supplement: Figure S2 — Validation of microarray data performed using GeneSifter analysis tool and its comparison with qPCR analysis. (A, B) Comparison of gene expressions fold changes between microarray and qPCR analyses for selected genes in granulosa cells collected from the ovulating follicle. Mean ± SEM fold expression changes −2 vs. 1 h (A) and −2 vs. 22 h (B) for selected genes are represented in the bar diagram. (C) Correlation analysis between log ratios of expressions obtained from microarray and real-time qPCR analyses. Linear regression analysis was performed for selected differentially expressed genes for −2 vs. 1 h (left panel), −2 vs. 22 h (right panel); using log10-transformed qPCR relative expression values (2−ΔΔCT; Y-axis) and log10-transformed fold change expression values obtained by microarray analysis (X-axis). P value indicates the significance of the correlation as determined by F test. R, correlation coefficient generated for the theoretical line of best fit (represented as solid line in each panel). (D) Immunolocalization of FKHR in granulosa cells from follicles in response to gonadotropin surge −2 and at 22 h post LH peak surge. Immunolocalization was performed using antibody specific to FKHR (A–H), after probing signal visualized using FITC-conjugated ant-rabbit IgG. Sections were counterstained with Hoechst to localize nuclei. Data is representative of three independent experiments. Pictures shown in panels A–C and E–G are of the same magnification (63×) and that of panels D and H are enlarged to higher zoom. Bar: 5 µm. (TIFF) [file pone.0020754.s002.tiff]

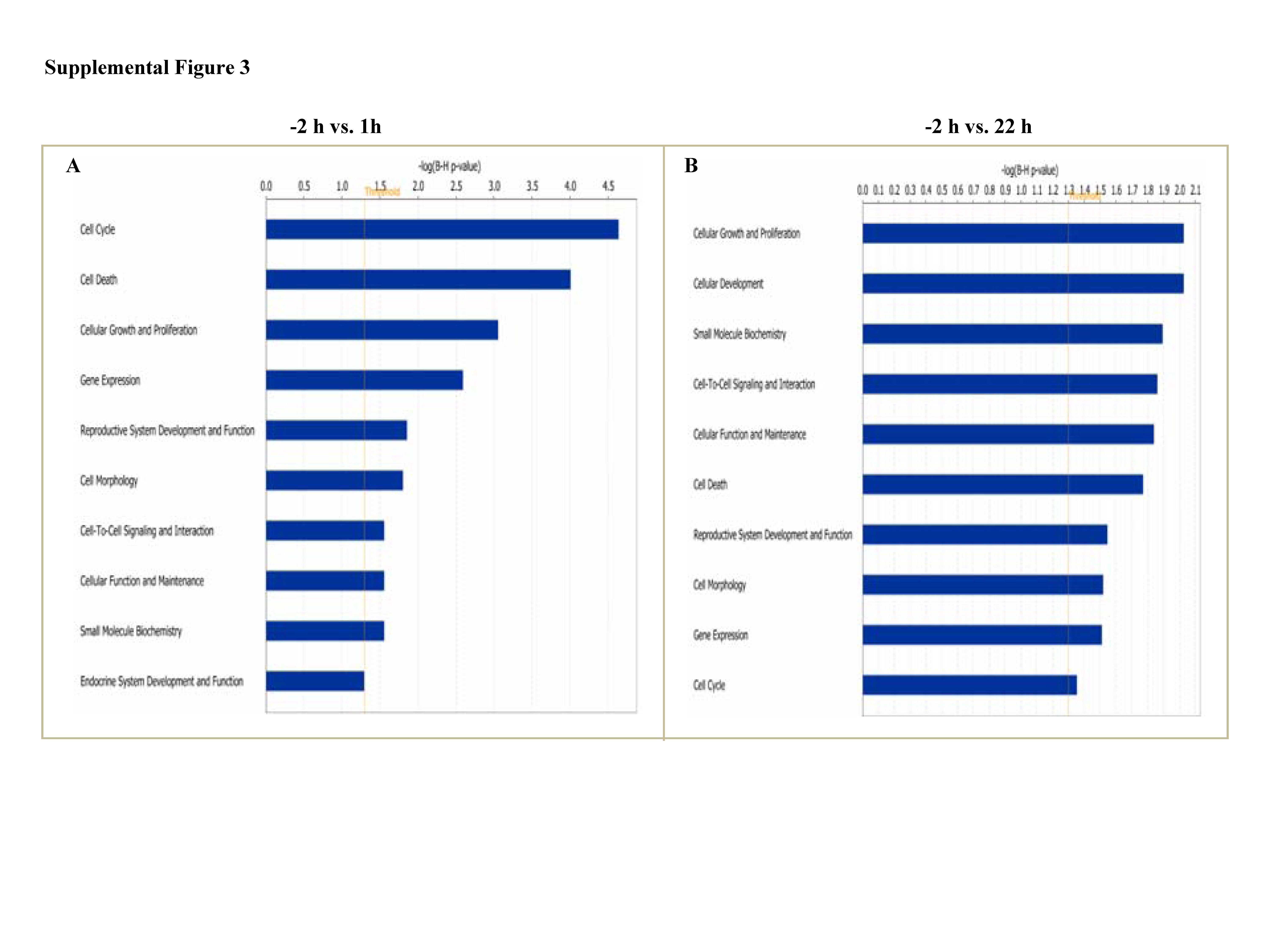

Supplement: Figure S3 — Ingenuity pathway analysis of differentially expressed genes into various ontological groups. Ingenuity pathway analysis classification of differentially expressed genes based on top 14 molecular and cellular functions, most significantly affected post peak LH surge in the granulosa cells. The blue bars indicate the likelihood [−log (B–H p-value)] that the specific ontological classification category was affected 1 (A) and 22 h (B) post peak LH surge compared with −2 h prior to peak LH surge. The threshold cut-off (orange line) is shown at p<0.05 (1.301 log scale). (TIFF) [file pone.0020754.s003.tiff]

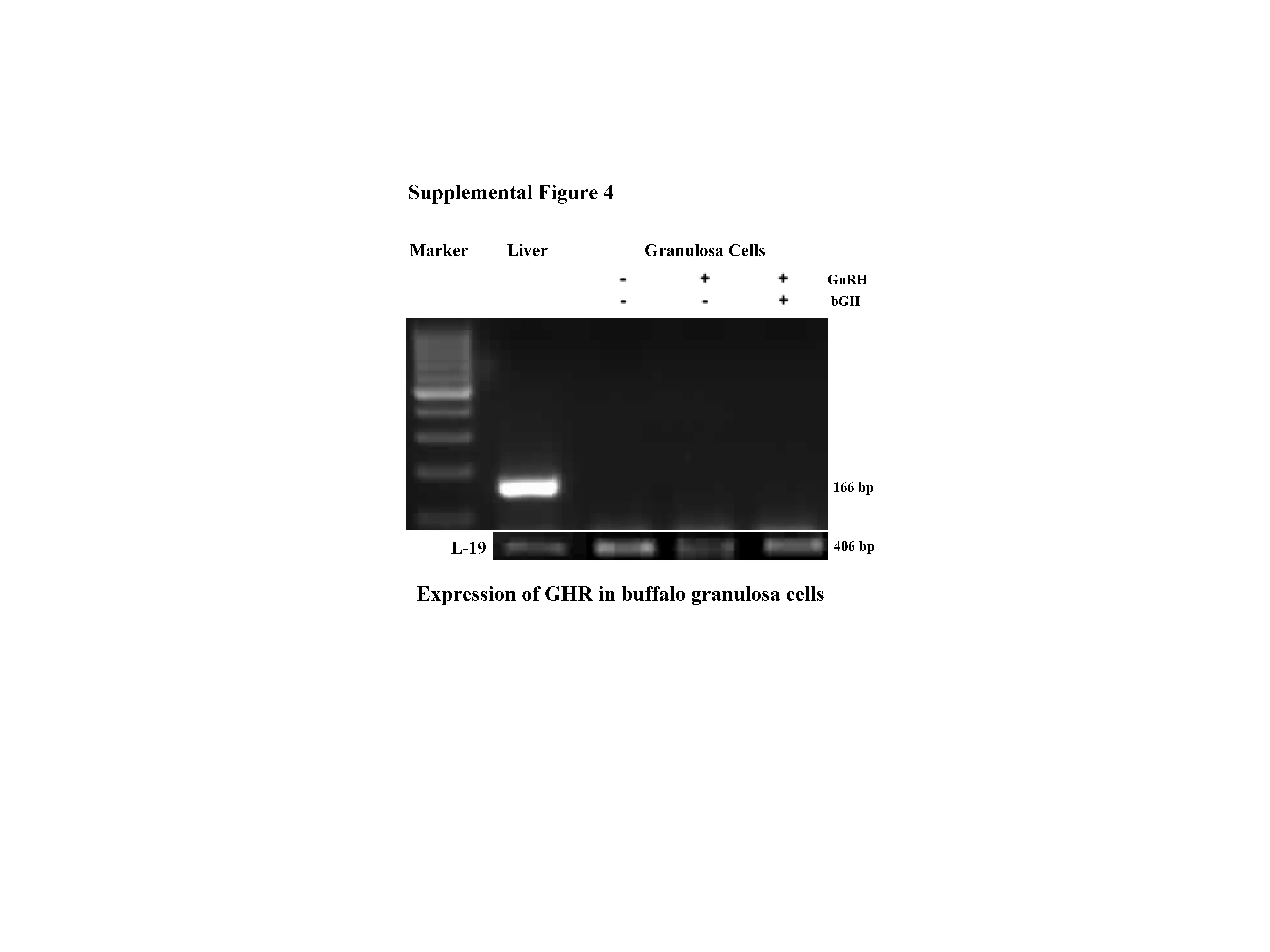

Supplement: Figure S4 — Expression of GHR mRNA in liver and granulosa cells of buffalo cows. Semi-quantitative RT-PCR analysis of GHR was performed in GCs collected from buffalo cows before and at 24 h post GnRH administration in animals treated with or without bGH. Total RNA (500 ng) isolated from liver and GCs was reverse transcribed and cDNA equivalent of 25 ng of total RNA was used for PCR reactions. The housekeeping gene, L19, was used as internal control. Shown here is a representative gel picture of GHR and L-19 PCR amplification products. (TIFF) [file pone.0020754.s004.tiff]

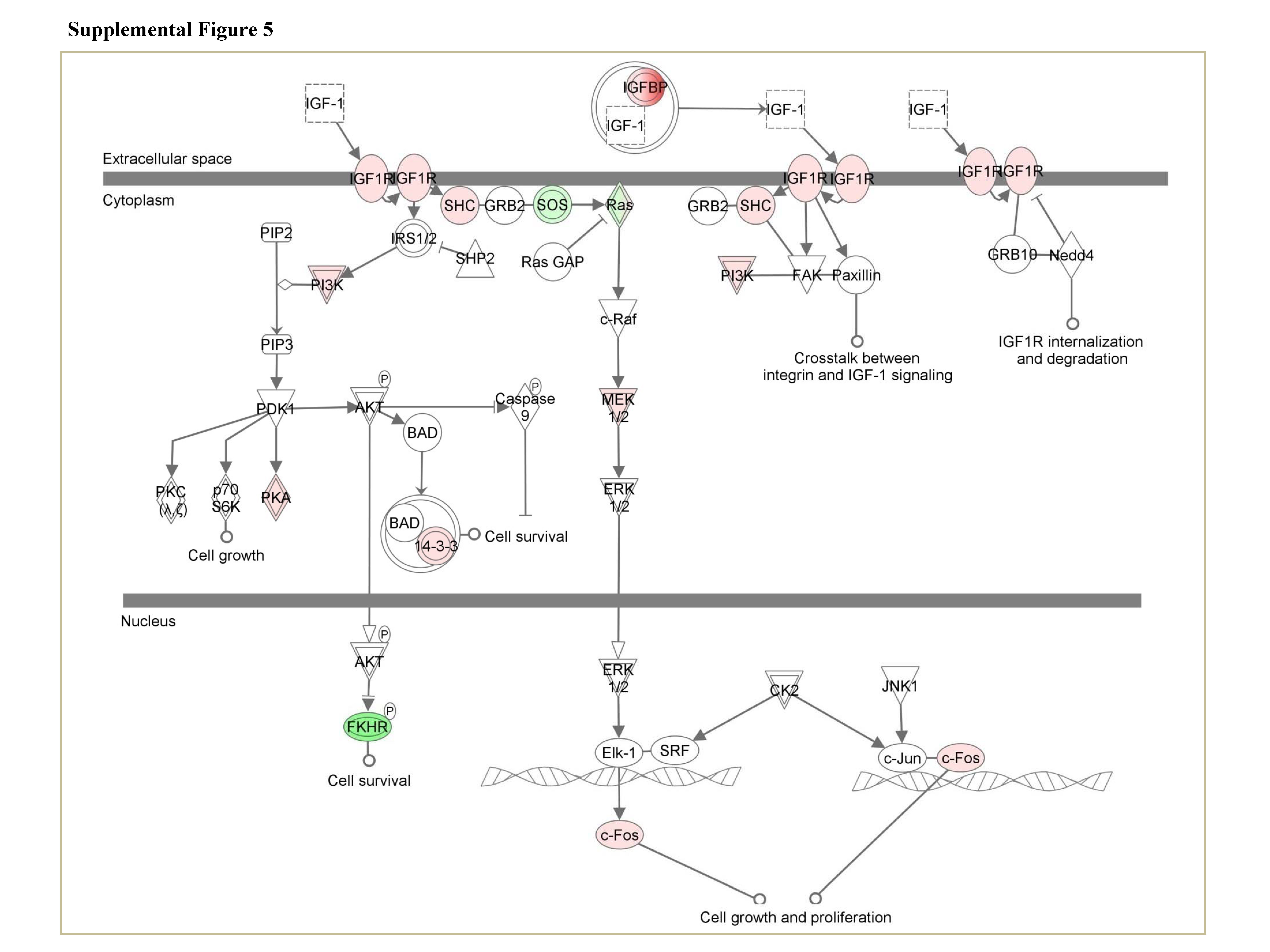

Supplement: Figure S5 — IGF-1 signaling pathway generated by Ingenuity pathway analysis software. The two canonical pathways, PI3K/AKT signaling and IGF-1 signaling pathways were the major biological functions associated with genes differentially expressed post gonadotropin surge. Each node represents a protein and the shape of node indicates functional class that may be up regulated (red) or down regulated (green) in the differentially expressed genes dataset, while proteins in open nodes were not found to be regulated in the microarray dataset. (TIFF) [file pone.0020754.s005.tiff]

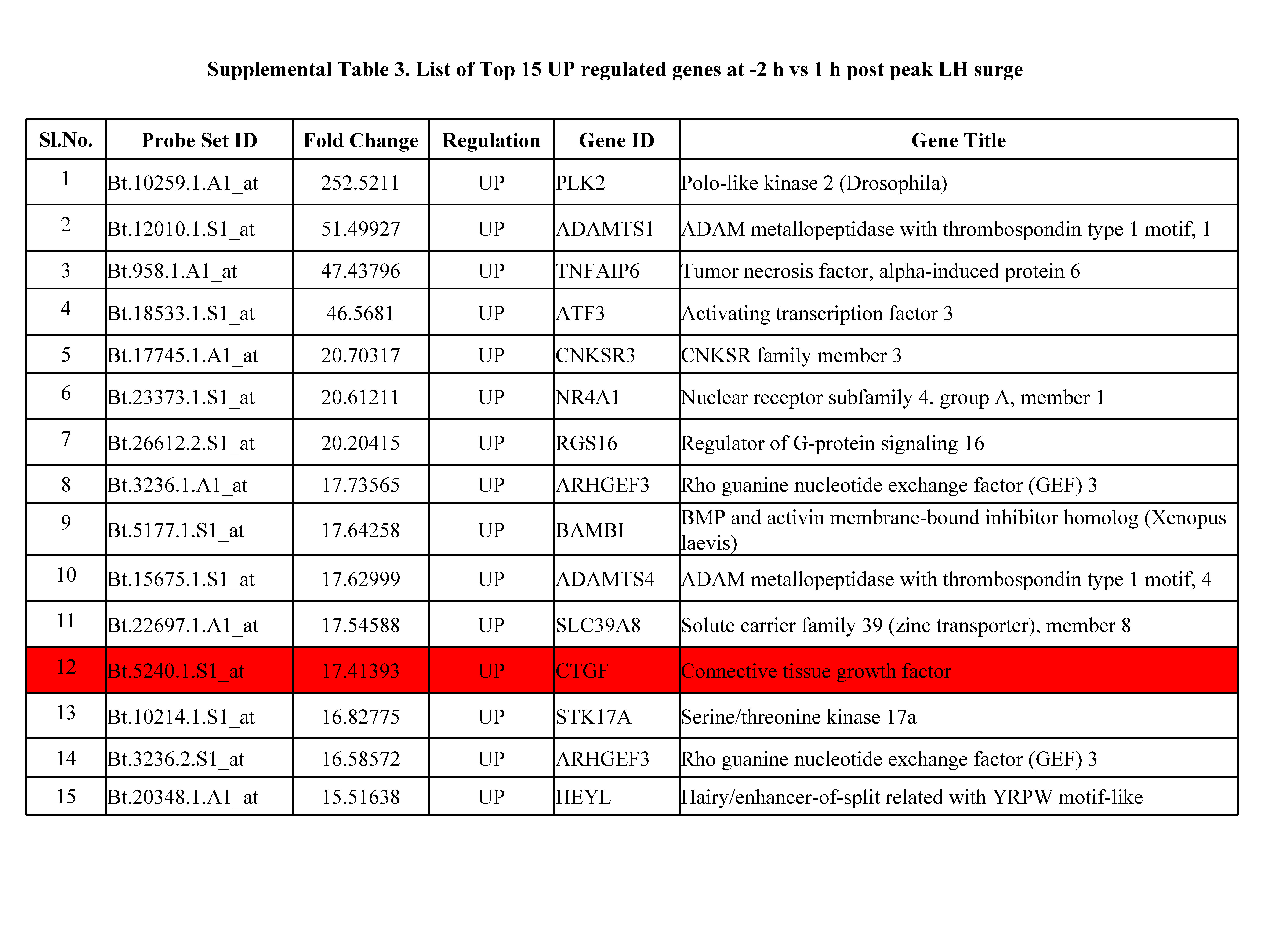

Supplement: Table S3 — List of top 15 UP regulated genes at −2 h vs 1 h post peak LH surge. Microarray data analysis was carried out to obtain a set of differentially expressed genes based on statistics, a t-test with p<0.05 and multiple hypothesis testing (Benjamini and Hochberg comparison test) to reduce the false positives. The identified differentially expressed genes that passed the statistical filters were further selected based on their presence in 80% of the samples examined and then another fold change cut-off filter use employed to narrow down the list. Microarray analysis was preformed with ≥2 fold change as cut-off for identification of differentially expressed genes. Whereas, the top 15 differentially UP regulated genes at 1 h post peak LH surge are represented in this table. Probe Set ID: The identifier that refers to a set of probe pairs selected to represent expressed sequences on an array; Fold Change: It is a number describing changes in expression level of a gene compared between control and treatment; Regulation: The expression of a particular gene in treated sample compared to control sample; Gene ID: gene symbols extracted from Entrez Gene or UniGene; Gene Title: gene name extracted from Entrez Gene or UniGene. The genes validated and analyzed further in the study are highlighted in red and others are discussed in the results section. (TIFF) [file pone.0020754.s012.tiff]

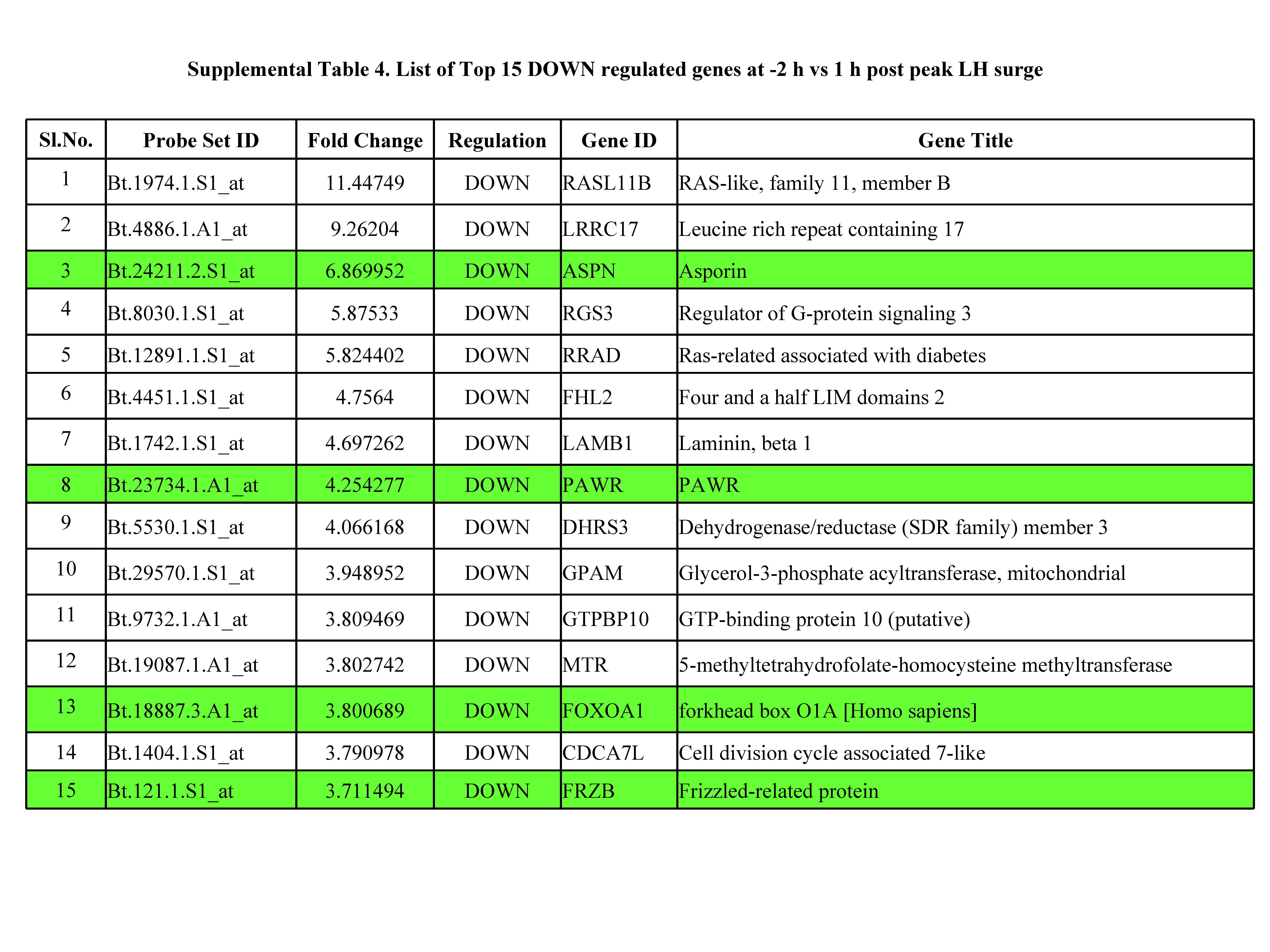

Supplement: Table S4 — List of top 15 DOWN regulated genes at −2 h vs 1 h post peak LH surge. The top 15 differentially DOWN regulated genes at 1 h post peak LH surge are represented. The list of genes validated and analyzed further in the study is highlighted in green, while the remaining genes are discussed in the results section. (TIFF) [file pone.0020754.s013.tiff]

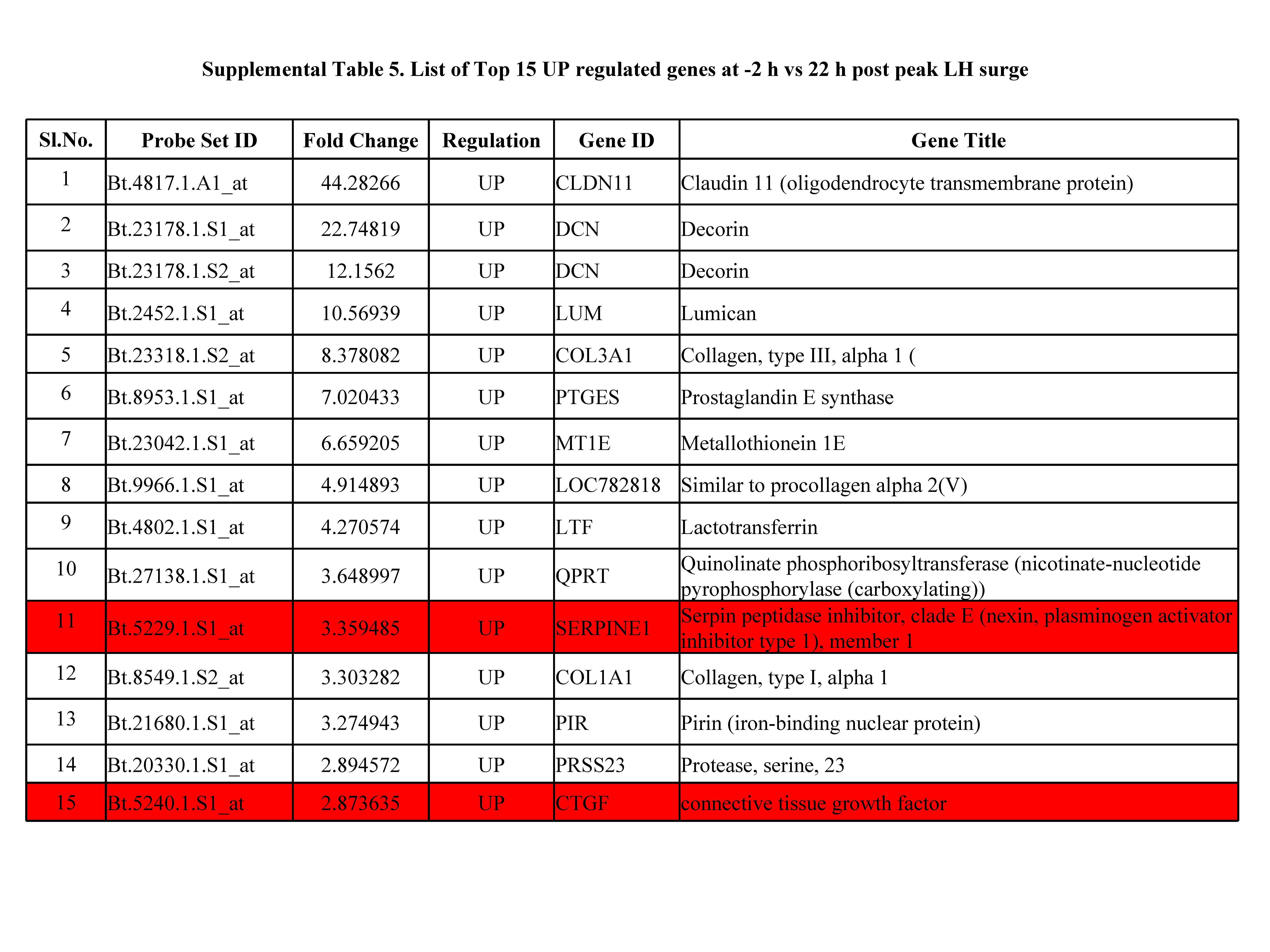

Supplement: Table S5 — List of top 15 UP regulated genes at −2 h vs 22 h post peak LH surge. The top 15 differentially UP regulated genes at 22 h post peak LH surge are represented. The list of genes validated and analyzed further in the study is highlighted in red, while the remaining genes are discussed in the results section. (TIFF) [file pone.0020754.s014.tiff]

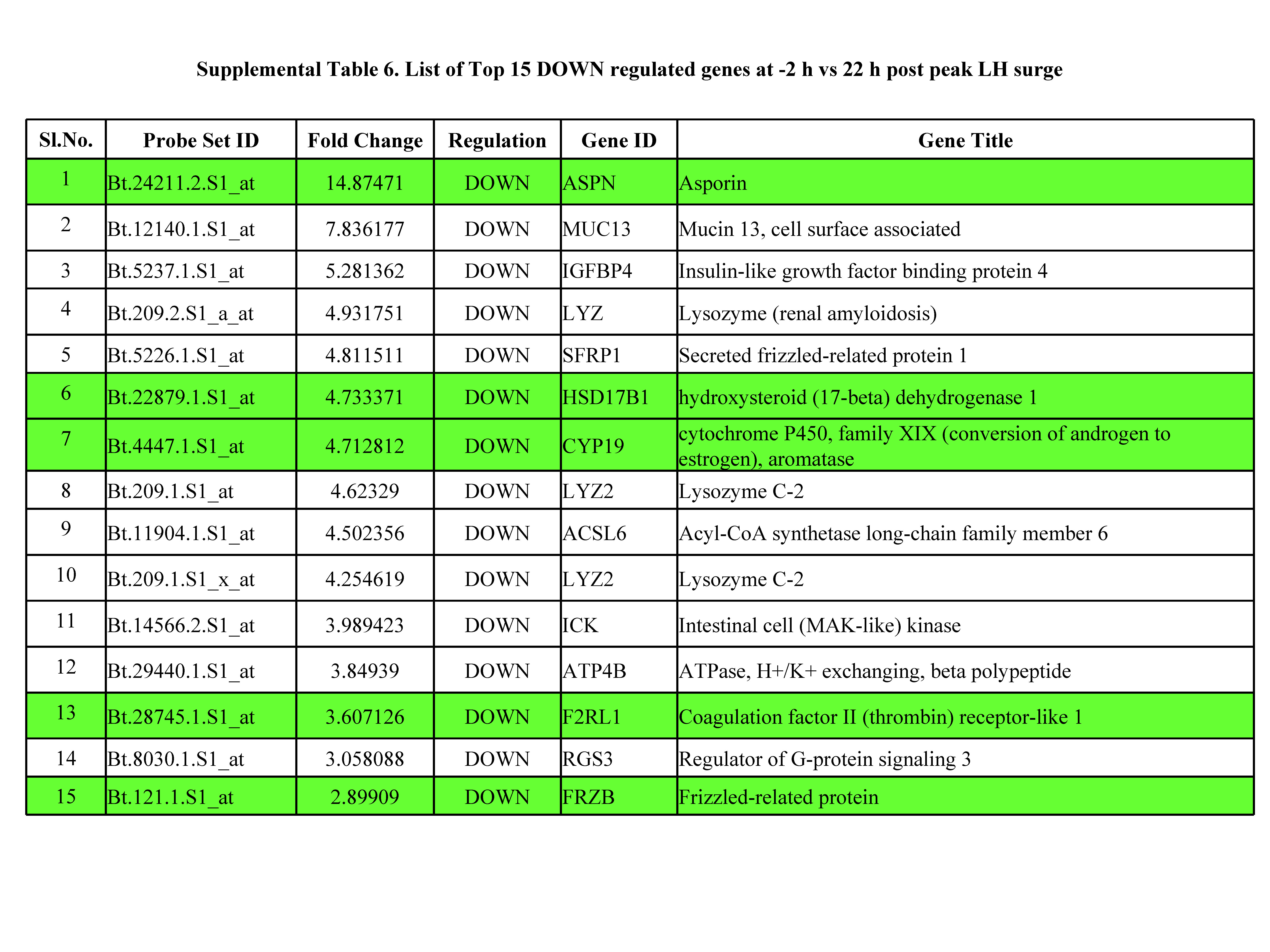

Supplement: Table S6 — List of top 15 DOWN regulated genes at −2 h vs 22 h post peak LH surge. The top 15 differentially DOWN regulated genes at 22 h post peak LH surge are represented. The list of genes validated and analyzed further in the study is highlighted in green and the remaining genes are discussed in the results section. (TIFF) [file pone.0020754.s015.tiff]
