# Supplementary material for: Gene Expression Profiling of Preovulatory Follicle in the Buffalo Cow: Effects of Increased IGF-I Concentration on Periovulatory Events
Source: PLoS One. 2011 Jun 20;6(6):e20754. doi: 10.1371/journal.pone.0020754 (PMC3119055; doi:10.1371/journal.pone.0020754)
Supplement: Data S1 — Validation of the experimental model. The results of validation of experimental model system employed for studying gene expression profiling of the ovulating follicle has been provided. (DOC) [file pone.0020754.s006.doc]

**Validation of the experimental model**

In buffalo cows, much similar to cattle, follicular growth occurs in two or three waves during estrous cycle [1,2]. Precise monitoring of the growth and development of follicles is necessary for time series analysis of events that unfold in the ovulatory follicle prior to and after occurrence of the gonadotropin surge. In bovine species, unlike the rodents, the onset of LH surge during spontaneous estrous cycle is not entrained to a particular day or time of the reproductive cycle. Moreover, the follicle selection results in selection of a single dominant follicle that needs to be accurately identified. In cattle extensive data exist on the success of various estrus and/or ovulation synchronization protocols employed for monitoring follicular growth [3]. Employing buffalo cows, we recently reported validation of experimental model in which the large follicle identified during the first wave of follicular growth was successfully induced to undergo ovulation following termination of corpus luteum function on day 7 of estrous cycle [4]. For the experiments described in this study, we validated a model system in which GnRH treatment was employed to stimulate endogenous gonadotropin surge and ovulation. The standardization of gonadotropin assays and dose of GnRH required for ovulation in the buffalo cow established for this study are described here briefly. On day 7 of estrous cycle after confirming for presence of at least one large follicle by ultrasound examination, four animals were administered Juramate 500 μg i.m. and 36 h later, animals were administered 100 μg of GnRH, i.m., blood samples were collected at 12 h intervals before GnRH injection and at hourly intervals for 6 h for monitoring circulating gonadotropins. For examining the characteristics of the ovulating follicle (expressions of genes and steroid levels for determining the ovulating status of follicle), ovaries were collected at slaughter from two animals 24 h post GnRH injection. In the other two animals, ultrasound examination was carried out at 2 h intervals for 8 h beginning 24 h post GnRH injection for evidence of ovulation (as judged by the disappearance of follicular contours). Also, to monitor circulating P_4_ concentrations, blood samples were collected on day 3, 5 and 7 post GnRH treatment. Circulating LH concentrations were low before GnRH injection, but rose quickly to reach peak concentration at 2 h (27.2 ± 4.5 ng/ml) before declining to pretreatment concentrations 5-6 h post injection. The expression of genes such as COX2, oxytocin and PR verified that the follicles had attained ovulatory status and in the two animals monitored for ovulation, the follicle contents disappeared 28 h after GnRH treatment and in both these animals, P_4_ concentrations progressively increased confirming formation of new CL. The results from this study in buffalo cows are in agreement with cattle [5,6]. This experimental model system was employed for studying gene expression profiling of the ovulating follicle and characterizing the effects of IGF-I on the ovulating follicle.

1. Baruselli PS, Mucciolo RG, Visintin JA, Viana WG, Arruda RP, et al. (1996) Ovarian follicular dynamics during the estrus cycle in buffalo (Bubalus bubalis). Preliminary research. Ann N Y Acad Sci 791: 408-411.

2. Manik RS, Palta P, Singla SK, Sharma V (2002) Folliculogenesis in buffalo (Bubalus bubalis): a review. Reprod Fertil Dev 14: 315-325.

3. Thatcher WW, Santos JE (2007) Control of ovarian follicular and corpus luteum development for the synchronization of ovulation in cattle. Soc Reprod Fertil Suppl 64: 69-81.

4. Jyotsna UR, Medhamurthy R (2009) Standardization and validation of an induced ovulation model system in buffalo cows: Characterization of gene expression changes in the periovulatory follicle. Anim Reprod Sci 113: 71-81.

5. Komar CM, Berndtson AK, Evans AC, Fortune JE (2001) Decline in circulating estradiol during the periovulatory period is correlated with decreases in estradiol and androgen, and in messenger RNA for p450 aromatase and p450 17alpha-hydroxylase, in bovine preovulatory follicles. Biol Reprod 64: 1797-1805.

6. Haughian JM, Ginther OJ, Kot K, Wiltbank MC (2004) Relationships between FSH patterns and follicular dynamics and the temporal associations among hormones in natural and GnRH-induced gonadotropin surges in heifers. Reproduction 127: 23-33.
