# Supplementary material for: Gene Expression Profiling of Preovulatory Follicle in the Buffalo Cow: Effects of Increased IGF-I Concentration on Periovulatory Events
Source: PLoS One. 2011 Jun 20;6(6):e20754. doi: 10.1371/journal.pone.0020754 (PMC3119055; doi:10.1371/journal.pone.0020754)
Supplement: Data S2 — Information necessary for MIAME compliance. The data provides the Minimum Information About a Microarray Experiment that is required to enable the interpretation of the results of the experiment unambiguously and potentially to reproduce the experiment. (PDF) [file pone.0020754.s007.pdf]

MIAME Checklist

Experimental Design

Authors:

Jyotsna UR, Kunal BS, Jayaram P and Medhamurthy R*
Department of Molecular Reproduction, Development and Genetics, Indian Institute of Science, Bangalore-560012, India
*Corresponding author: **R Medhamurthy** Email: **rmm@mrdg.iisc.ernet.in**

Type of experiment:

Time course experiment, the objective of this study is to analyze gene expression changes 1 and 22 h post peak LH surge in the granulosa cells of the buffalo cow.

Experimental details:

Ovaries were collected at slaughter before, 3, 10 and 24 h post GnRH injection (n=4 animals or more/time point), following confirmation of time of peak LH surge, these time points correspond to -2, 1, 10 and 22 h post peak LH surge. These time points were chosen with a view to catalogue gene expression changes in the preovulatory follicle at the earliest time point (i.e., 1 h) and at a later time point (i.e., 22 h) post peak LH surge the time point closest to the time of ovulation.

Number of hybridizations:

9 hybridizations on oligonucleotide arrays (Affymetrix Bovine Genome Array)

Hybridization design:

Affymetrix system, single colour

Quality control steps:

Standard Affymetrix control steps have been employed in the present study. Since the quantity of total RNA obtained from GCs in each animal was less, a linear two step amplification method using protocols recommended by Affymetrix GeneChip® expression analysis technical manual was employed. The complete details of the amplification protocol are provided in the materials and methods section of the manuscript. Approximately 200 ng of each biotinylated sample cRNA target along with a biotinylated control cRNA target was analyzed on the RNA 6000 LabChip using the 2100 Bioanalyzer (Agilent Technologies). The target quality was determined based on cRNA yield and size distribution produced from the in vitro synthesis reaction. Samples that failed quality control were discarded or relabeled.

Number of replicates:

9 biological replicates: n = 3 per time point, 3 time points in total (-2, 1 and 22 h post peak LH surge).

URL of web site / database accession number submitted at GEO:

The raw data and the related data files discussed in this publication have been deposited at NCBI's Gene Expression Omnibus (http://www.ncbi.nlm.nih.gov/gds?term=GSE11312) and are accessible through GEO Series accession number GSE11312.

Samples

Origin of sample:

Ovaries were collected at slaughter from the experimental non lactating buffalo cows

Manipulation of samples:

Ovaries containing preovulatory follicles were processed for retrieval of GCs and follicle wall, no other manipulations or treatments performed.

Protocol for preparing hybridization extract:

Total RNA was isolated from GCs using TRI reagent (Sigma Aldrich Corporation, Bangalore, India) followed by a cleaning step using the RNeasy mini kit for RNA purification following manufacturer recommendations. Since the quantity of total RNA obtained from GCs in each animal was less, a linear two step amplification method using protocols recommended by Affymetrix GeneChip® expression analysis technical manual was employed.

Labelling protocol:

The entire procedure of microarray target preparation, hybridization and scanning was performed at Center for Integrated Biosystems, Utah State University, Utah. The complete details of the amplification and labelling protocol are provided in the materials and method section of the manuscript.

External controls (spikes):

Biotinylated hybridization control oligomer and biotinylated control cRNAs for BioB, BioC, BioD and CreX (Affymetrix) at 1.5, 5, 25, and 100 pM concentrations, respectively, in hybridization buffer.

Hybridization Procedures and Parameters

Protocol and conditions:

Protocols recommended by Affymetrix GeneChip® expression analysis technical manual were employed.
http://media.affymetrix.com/support/downloads/manuals/expression_analysis_technical_manual.pdf

Measurement Data and Specifications

Type of scanning hardware and software used:

Software – GCOS version 1.4.0 (Affymetrix)
Scanning hardware – GeneChip Scanner 3000 with 7G upgrade (Affymetrix)

Type of image analysis software used:

GCOS version 1.4.0 (Affymetrix)

Description of measurements produced by the image-analysis software and measurements used in the analysis:

Probe level measurements produced by GCOS version 1.4.0 (.cel files).

Complete output of the image analysis before data selection and transformation (spot quantitation matrices):

Original Affymetrix output files (.cel and .chp files)

Data selection and transformation procedures:

The Robust Multichip Analyis (RMA) was used for signal quantification and performed using GeneSifter software (www.genesifter.com). The RMA was applied to the probe-level measurements contained in .cel files. Data analysis was performed using GeneSifter.

Final gene expression data table(s) used by authors to make their conclusions after data selection and transformation (gene expression data matrices):

1. Control (2 h before peak LH surge) vs Treatment (1 h post peak LH surge) comparisons (-2 h vs 1 h.xls supplemental file 11)
2. Control (2 h before peak LH surge) vs Treatment (22 h post peak LH surge) comparisons (-2 h vs 22 h.xls supplemental file 12)

Array Design

Platform type:

Affymetrix Oligonucleotide Array

Surface and Coating Specifications:

Glass

Array:

Affymetrix Bovine Genome Array

Features on the array:

See http://media.affymetrix.com/support/technical/datasheets/bovine_datasheet.pdf
